# Calcineurin Regulates Conidiation, Chlamydospore Formation and Virulence in *Fusarium oxysporum* f. sp. *lycopersici*

**DOI:** 10.3389/fmicb.2020.539702

**Published:** 2020-10-22

**Authors:** Yi-Hsuan Hou, Li-Hang Hsu, Hsuan-Fu Wang, Yu-Hsin Lai, Ying-Lien Chen

**Affiliations:** Department of Plant Pathology and Microbiology, National Taiwan University, Taipei, Taiwan

**Keywords:** *Fusarium oxysporum*, calcineurin, conidiation, chlamydospore, virulence

## Abstract

Fusarium wilt of tomato caused by the ascomycetous fungus *Fusarium oxysporum* f. sp. *lycopersici* (*Fol*) is widespread in most tomato planting areas. Calcineurin is a heterodimeric calcium/calmodulin-dependent protein phosphatase comprised of catalytic (Cna1) and regulatory (Cnb1) subunits. Calcineurin has been studied extensively in human fungal pathogens, but less is known about its roles in plant fungal pathogens. It is known that calcineurin regulates fungal calcium signaling, growth, drug tolerance, and virulence. However, the roles of calcineurin in *Fol* have not yet been characterized. In this study, we deleted calcineurin *CNA1* and *CNB1* genes to characterize their roles in conidiation, chlamydospore formation and virulence in *Fol*. Our results revealed that both *cna1* and *cnb1* mutants show defects in calcineurin phosphatase activity, vegetative growth and conidiation as compared to the wild type. Furthermore, calcineurin mutants exhibited blunted and swollen hyphae as observed by scanning electron microscopy. Interestingly, we found that *Fol* calcineurin is critical for chlamydospore formation, a function of calcineurin previously undocumented in the fungal kingdom. According to transcriptome analysis, the expression of 323 and 414 genes was up- and down-regulated, respectively, in both *cna1* and *cnb1* mutants. Based on the pathogen infection assay, tomato plants inoculated with *cna1* or *cnb1* mutant have a dramatic reduction in disease severity, indicating that calcineurin has a vital role in *Fol* virulence. In conclusion, our findings suggest that *Fol* calcineurin is required, at least in part, for phosphatase activity, vegetative growth, conidiation, chlamydospore formation, and virulence.

## Introduction

Calcineurin, a calcium/calmodulin-dependent serine/threonine protein phosphatase, which forms a heterodimer comprised of a catalytic A subunit (Cna) and regulatory B subunit (Cnb) is conserved in eukaryotes (Juvvadi et al., 2014). In the fungal kingdom, calcineurin is responsible for maintaining a diverse range of cellular processes such as ion homeostasis, growth, morphogenesis, stress response, and pathogenicity by activating downstream events (Park et al., 2019). In general, when fungal cells encounter external stress, the plasma membrane or cell compartment Ca^2+^ influx system will be activated, resulting in an increased intracellular Ca^2+^ concentration. In the calcineurin cascade, calmodulin (CaM) and regulatory B subunit of calcineurin act as a sensor for Ca^2+^ signals, then Ca^2+^/CaM will specifically bind to the catalytic A and regulatory B subunits of calcineurin to form a Ca^2+^/CaM-calcineurin complex, leading to functional phosphatase activity (Stemmer and Klee, 1994). The activated calcineurin subsequently dephosphorylates the downstream targets, such as Crz1 and Prz1, allowing their nuclear import and inducing the expression of target genes (Juvvadi and Steinbach, 2015).

In baker’s yeast *Saccharomyces cerevisiae*, calcineurin is required for adaptation and growth under environmental stress and at higher alkaline pH (Cyert, 2003). Previous reports revealed that calcineurin plays roles in stress response and growth at mammalian body temperature (37°C) in *Cryptococcus neoformans* (Park et al., 2016). Similarly, in *Candida* species, calcineurin is required for drug tolerance, virulence and survival in serum (Yu et al., 2015; Park et al., 2019). Moreover, calcineurin is essential for proper hyphal growth in the filamentous fungus *Aspergillus fumigatus* (Juvvadi et al., 2011), and is necessary for cell cycle progression in *Aspergillus nidulans* (Rasmussen et al., 1994).

By using RNAi strategies, the role of the catalytic subunit Cna1 was characterized in plant fungal pathogen *Sclerotinia sclerotiorum* and was found to be critical for sclerotial development (Harel et al., 2006). In *Magnaporthe oryzae*, Cna1 was found to be involved in appressorium formation, conidiation, and hyphal growth (Choi et al., 2009). Meanwhile, Cna1 plays roles in ear gall formation and morphogenesis of *Ustilago maydis* (Egan et al., 2009). In *Ustilago hordei*, null mutation in either the catalytic or regulatory subunit resulted in sensitivity to environmental stresses and severely reduced virulence in barley plants (Cervantes-Chavez et al., 2011). In addition, the calcineurin signaling pathway was involved in the morphogenetic differentiation and haustoria formation in *Puccinia striiformis* f. sp. *tritici* (Zhang et al., 2012).

However, the roles of calcineurin signaling have not been reported in the important plant fungal pathogen *Fusarium oxysporum* f. sp. *lycopersici*, a devastating pathogen that causes tomato wilt. Fusarium wilt of tomato, a soil-borne disease, is one of the limiting factors in tomato yield. *Fol* can produce three kinds of asexual spores including microconidia, macroconidia and chlamydospore, and these infectious propagules play critical roles in the disease cycle (Agrios, 2005). In *Fol*, formation of chlamydospores serves as a survival strategy during undesirable conditions. Once in favorable weather, chlamydospores will act as primary inocula to invade tomato roots, clog water flow and nutrient movement by colonizing the vascular bundles, leading to yellowed leaves and wilted plants, and eventually decreased yield of tomato. Meanwhile, the macroconidia and microconidia can be produced from stem surfaces and leaves of *Fol* infected tomato, which in turn serve as the secondary inocula, thereafter infect neighboring healthy plants by spreading manner (Pietro et al., 2003; Manikandan et al., 2018).

In the present study, we characterized calcineurin function through deletion of the *CNA1* and *CNB1* genes in *Fol*. Our results revealed that the calcineurin has vital functions in conidiation, chlamydospore formation, and virulence in *Fol*.

## Materials and Methods

### Strains, Media and Chemicals

*F. oxysporum* f.sp. *lycopersici* (*Fol*) 4287 wild type (WT) and calcineurin mutants (Table 1) were grown on potato dextrose agar (PDA) (0.4% potato starch from infusion, 2% dextrose, 1.5% agar) (BioShop, Burlington, ON, Canada). A soil medium made with 200 g horticultural substrate (Kekkila Finnish peat moss), 0.2 g CaCO_3_, 0.5% glucose and 700 mL ddH_2_O (filtered after autoclaved) was used for chlamydospore production (Chen et al., 2015). For the pharmacological inhibition test of calcineurin, PDA medium containing calcineurin inhibitor FK506 (Astellas Pharma, Tokyo, Japan) or cyclosporine A (LC Laboratories, Woburn, United States) were used. The growth temperature for all fungal strains was set at 25°C.

**TABLE 1:** *Fusarium oxysporum* f. sp. *lycopersici* strains used in this study.

**Strain**	**Genotype**	**Parent**	**Reference**
4287	Prototrophic wild type		Ma et al., 2010
HFW1	Δ*cna1:*P*gpdA*-*hyg*^R^-*gfp*-T*trpC*	4287	This study
LHS2	Δ*cna1:*P*gpdA*-*hyg*^R^-*gfp*-T*trpC*	4287	This study
HFW3	Δ*cnb1:*P*gpdA*-*hyg*^R^-*gfp*-T*trpC*	4287	This study
HFW4	Δ*cnb1:*P*gpdA*-*hyg*^R^-*gfp*-T*trpC*	4287	This study
*cna1*:*CNA1* (YHH1)	Δ*cna1:CNA1*	HFW1	This study
*cnb1*:*CNB1* (YHH2)	Δ*cnb1:CNB1*	HFW3	This study

### Generation of Calcineurin Mutants and Complementary Strains

To disrupt the *FolCNA1* gene, 5′ and 3′ non-coding region (NCR) of *FolCNA1* were PCR amplified from genomic DNA of *Fol* using primer sets JC753/JC768 (5′ NCR of *FolCNA1*, 618 bp) and JC769/JC756 (3′NCR of *FolCNA1*, 546 bp). Primers JC766/JC767 were used to amplify the hygromycine B resistance gene (*hyg*^*R*^) from the plasmid pPK2-hphgfp (Punt et al., 1987), which also contained GFP (*gfp*) under control of the *A. nidulans* gpdA promoter (P*gpd*A) and trpC terminator (T*trp*C). These PCR products were treated with ExoSAP-IT (USB Corp., Ohio, United States) to remove additional primers and dNTPs. The *FolCNA1* gene disruption cassette (5848 bp) was generated by fusion PCR of these three PCR fragments with primers JC753/JC756 (Supplementary Figure S3A). The same approach was employed to generate *FolCNB1* deletion mutants; the 5′ and 3′ non-coding region (NCR) of *FolCNB1* were PCR amplified from genomic DNA of *Fol* using primer sets JC839/JC840 (5′ NCR of *FolCNB1*, 514 bp) and JC841/JC842 (3′NCR of *FolCNB1*, 521 bp). The *FoCNB1* gene disruption cassette (5654 bp) was generated by fusion PCR of the DNA fragments of 5′ and 3′ NCR of *FolCNB1* and *hyg*^*R*^ using primers JC839/JC842 (Supplementary Figure S3B).

The *FolCNA1* and *FolCNB1* gene disruption cassettes were introduced into *Fol* wild type 4287 using the protoplast transformation method to generate *cna1* and *cnb1* deletion mutants (Moradi et al., 2013). Transformants were confirmed by PCR using primer sets JC788/JC961, JC772/JC773, and JC922/JC827 for *cna1* mutants, and JC922/JC961, JC843/JC844, and JC922/JC903 for *cnb1* mutants, to identify whether the targeted DNA is successfully recombined and replaced. The observation of GFP fluorescence was also conducted to confirm those mutants (Supplementary Figure S2A). Two independent mutants of *cna1* (HFW1 and LHS2) and *cnb1* (HFW3 and HWF4) were obtained in this study.

For complementation analysis of the *cna1* mutant, a full length of *FolCNA1* genomic DNA containing 5′NCR plus *CNA1* open reading frame (ORF), and its 3′ NCR were PCR amplified from genomic DNA of *Fol* by using primer sets JC753/JC838 (5′ NCR+ORF of *FolCNA1*, 3567 bp) and JC837/JC756 (3′NCR of *FolCNA1*, 526 bp), respectively. Moreover, primers JC836/JC767 were used to amplify the bleomycin resistance gene (*Bleo*^*R*^) from the plasmid pRW1 (Michielse et al., 2009a). These PCR products were treated with ExoSAP-IT (USB Corp., Ohio, United States) to remove additional primers and dNTPs. The reaction of fusion PCR containing three DNA fragments were carried out with primers JC753/JC756, to generate 6655 bp complementation cassette (*5′NCR-FolCNA1*-*Bleo*^*R*^-*3′NCR*) (Supplementary Figure S4A). The similar approach was used to generate the complementary strain of *cnb1* mutant; 5′ NCR plus *FolCNB1* ORF and 3′ NCR of *FolCNB1* were PCR amplified from genomic DNA of *Fol* using primer sets JC839/JC925 (5′ NCR+ORF of *FolCNB1*, 2093 bp) and JC841/JC842 (3′NCR of *FolCNB1*, 500 bp), respectively. The complementation cassette of *cnb1* mutant (*5′NCR-FolCNB1*-*Bleo*^*R*^-*3′NCR*, 5155 bp; Supplementary Figure S4A) was generated by fusion PCR containing DNA fragments of 5′ NCR+ *FolCNB1* and *Bleo*^*R*^ and the 3′ NCR of *FolCNB1* with using primers JC839/JC842. The *CNA1* and *CNB1* complementation cassettes were then introduced into *cna1* and *cnb1* mutant, respectively, using the protoplast transformation method. Transformants were confirmed by PCR using primer sets JC1404/JC1405 and JC772/JC773 for *cna1*:*CNA1* (YHH1); JC1404/JC1405 and JC843/JC844 for *cnb1*:*CNB1* (YHH2), to verify the expected DNA recombination in complemented strains (Supplementary Figure S4B).

### Colony Morphology Observation

The wild type and calcineurin mutants were streaked out from −80°C stock, cultured on PDA and incubated at 25°C for 2 weeks, and cut 5 mm in diameter with a hole punch. Agar disks containing cultures were put on PDA plates in the presence or absence of 1 μg/mL FK506 or 100 μg/mL cyclosporine A with three replicates. All plates were then incubated at 25°C for 7 days, and the diameters of fungal colonies were measured daily. Data were analyzed by two-way ANOVA and Bonferroni post-test using GraphPad Prism 5 (GraphPad Software, CA, United States).

### Chlamydospore Formation

Soil medium was used to trigger *Fol* strains to produce chlamydospores. Conidial suspension (500 μL) at a concentration of 5 × 10^5^ conidia/mL was inoculated into 100 mL soil medium, and agitated at 25°C for 7 days. Cultures filtered through miracloth, and mycelium-chlamydospore aggregates were resuspended in 20 mL ddH_2_O and homogenized with beads using a Geno/Grinder machine (SPEX SamplePrep) at 1700 rpm for 15 s.

For the morphological observation of the chlamydospore, mycelium-chlamydospore aggregates were stained with 0.3% Calcofluor white (CFW; Fluorescent Brightener 28; Sigma, St. Louis, United States) and Nile red (NR; Sigma, St. Louis, United States) for 5 min. After staining, chlamydospores were washed twice in sterile water and observed by fluorescence microscopy.

The concentration of chlamydospore was determined via a hemocytometer. Analyses were performed by one-way ANOVA and Dunnett’s post-test using GraphPad Prism 5. The aggregates were dried, and chlamydospores produced per gram of dried mycelia (spores/g) were calculated according to the formula shown below:

$$\frac{\begin{array}{c} Concentrationofchlamydospores\left(spores/mL\right)\times \\ T\,o\,t\,a\,l\,v\,o\,l\,u\,m\,e\,\left(20\,m\,L\right) \end{array}}{D\,r\,y\,w\,e\,i\,g\,h\,t\,o\,f\,m\,y\,c\,e\,l\,i\,a\,\left(g\right)}$$

### Calcineurin Activity Assay

To assay for calcineurin phosphatase activity, 500 μL conidia suspension of *Fol* wild type and calcineurin mutants at a concentration of 5 × 10^5^ conidia/mL were cultured in PDB medium (BioShop, potato dextrose broth: 0.4% potatoes from infusion) at 25°C, and agitated for 72 h. Mycelia were frozen in liquid nitrogen and ground into powder. Protein crude extracts were prepared by homogenizing the mycelia powder in a buffer containing 50 mM Tris-HCl (pH 7.4), 1 mM EGTA, 0.2% Triton X-100, 1 mM PMSF (Roche, San Francisco, CA, United States) and 1:100 protease inhibitor cocktail was added (Roche, San Francisco, CA, United States). The homogenates were clarified by centrifugation at 5,000 × *g* for 10 min at 4°C and then at 20,000 × *g* for 60 min at 4°C. Protein concentration in the extracts were determined by Bradford’s method (Bradford, 1976). Phosphatase activity of calcineurin was assayed according to methods described previously (Perrino et al., 1995; Juvvadi et al., 2011). In brief, the phosphatase activity assay was performed in a total volume of 100 μL containing reaction mixture [25 mM Tris (pH 7.2), 25 mM MES (pH 7.0), 1 mM MnCl_2_, 50 mM *p*-nitrophenyl phosphate] and homogenate. After incubation at 30°C for 10 min, the reaction was stopped by addition 10 μL of 13% (w/v) K_2_HPO_4_. The absorbance of the samples was measured at 405 nm via microplate spectrophotometer (Spectra MAX 190, Molecular Devices, CA, United States). The difference of absorbance values between the amounts of *p*-nitrophenol represented the phosphatase activity of calcineurin. The experiments were performed in triplicate, and the data were analyzed by one-way ANOVA and Dunnett’s post-test using GraphPad Prism 5. The calcineurin phosphatase activity was calculated according to the formula shown below:

$$\frac{\begin{array}{c} V\,o\,l\,u\,m\,e\,\,o\,f\,\,t\,h\,e\,\,r\,e\,a\,c\,t\,i\,o\,n\,\,\left(\mu \,L\right)\times O\,D^{405}\\ \left(c\,m^{-1}\right)\,\left(c\,o\,n\,i\,d\,i\,a/m\,L\right) \end{array}}{\begin{array}{c} pNPPmillimolarextintioncoefficient\left(18,000M^{-1}\cdot cm^{-1}\right)\times \\ I\,n\,c\,u\,b\,a\,t\,i\,o\,n\,t\,i\,m\,e\,\,\left(\text{min}\right)\times P\,r\,o\,t\,e\,i\,n\,\,c\,o\,n\,c.\left(\mu \,g\right) \end{array}}$$

### Microscopy Analysis

For scanning electron microscopy (SEM) observation, *Fol* strains were grown on PDA and incubated at 25°C for 2 weeks, and cut 5 mm in diameter with a hole punch. The agar disks were fixed with 4% glutaraldehyde for 3 h and treated with 0.1 M cacodylate for 1 h. The fixed mycelia were then dehydrated consecutively with 30, 50, 70, 85, 90, 95, and 100% ethanol for 20 min each. Final treatment was conducted with 100% acetone for 20 min, and the specimens were allowed to dry in critical-point dryer (Hitachi HCP-2, Japan) and then sputter coated with gold by ion-coater (Hitachi E101, Japan). The specimens were observed on a FE-SEM (FEI Inspect S, United States) using an accelerating voltage of 15 kV. These instruments and chemicals were provided by Technology Commons, College of Life Science, National Taiwan University.

For the observation of the hyphal compartment, strains were cultured in PDB and incubated at 25°C for 24 h. Mycelia were collected and stained with 0.3% Calcofluor white (CFW; Fluorescent Brightener 28; Sigma, St. Louis, United States) for 5 min. After staining, mycelia were washed twice in sterile water and observed by fluorescence microscopy. The average length between septum-to-septum was measured by randomly selecting 20 mycelia from each of three technical replicates. Considering of the apical growth of fungi, the hyphal tips and lateral branches were excluded in the measurement and each mycelium were fixed length at 200 μm.

### Conidial Germination Rate

In order to examine the germination rate, *Fol* strains were streaked out from −80°C stock and grown on PDA, incubated at 25°C for 2 weeks. Conidia were collected using ddH_2_O, filtrated through one-layer miracloth and washed several times. Conidial suspension was adjusted to a concentration of 5 × 10^5^ conidia/mL and incubated at 25°C with shaking at 250 rpm to keep the spores suspended. The germination rate was calculated by randomly counting 50 conidia from each of three replicates over 8 h, and germinated conidia were defined as those containing germ tubes longer than half the length of the conidia.

### Plant Infection Assay

The tomato cultivar *Solanum lycopersicum* Farmer 301 (Known-You Seed Co., Kaohsiung, Taiwan) was used in this study. Two-week-old tomato seedlings were inoculated by submerging roots in a suspension of 5 × 10^6^ conidia/mL of *Fol* wild type or calcineurin mutants for 30 min, and then transplanting into cultural substrate [vermiculite:perlite:peat moss (1:1:1)]. Tomato plants were grown in a green house under a 16/8 h light-dark photoperiod at 28°C. Disease severity was recorded at 21 dpi. Scales of disease index were graded into five degrees from 0 to 4: 0 = no symptom; 1 = one to three yellowing leaves but not withering; 2 = at least four yellowing leaves but only one to two withering; 3 = at least four yellowing leaves and three withering; and 4 = all vascular bundles are brown, plant either dead, very small or wilted. Ten plants were used for each treatment. Data were analyzed by one-way ANOVA and Dunnett’s post-test using GraphPad Prism 5. The formula of disease severity is shown below:

$$\frac{\begin{array}{c} \sum (numberofplantletsymptomatic\\ plants\times diseasegrade) \end{array}}{\begin{array}{c} totalnumberofdiseasescale\times \\ m\,a\,x\,i\,m\,u\,m\,d\,i\,s\,e\,a\,s\,e\,g\,r\,a\,d\,e \end{array}}\times 100\%$$

### RNA Sequencing Analysis

*Fol* wild type, and *cna1* (HFW1) and *cnb1* (HFW3) mutants were cultured in PDB at 25°C for 14 h. For total RNA extraction, mycelia were collected and frozen in liquid nitrogen, then ground into powder. The powder was transferred to a 1.5 mL centrifuge tube followed by adding an equal volume of TRIzol reagent (Invitrogen, Carlsbad, CA, United States) and 100 μL chloroform, mixed well and centrifuged at 14,000 rpm for 5 min at 4°C. The upper phase was collected and precipitated with isopropanol by centrifuging at 14,000 rpm for 10 min at 4°C. The supernatants were discarded, and the nucleic acid pellets were serially washed in 70 and 100% ethanol. The RNA was then re-suspended with DEPC water.

RNA sequencing was performed by Genomics BioSci &Tech. Co., Ltd, New Taipei City, Taiwan. The RNA quality analysis was conducted using Agilent 2100 Bio-analyzer and Real-Time PCR system, to confirm that the RNA samples had a RIN value ≥ 7. The oligo(dT)-attached beads were used to purify mRNA, which was fragmented for cDNA synthesis. The first strand of cDNA was synthesized by reverse transcriptase with a random primer, and double strand cDNA was synthesized by using dUTP in place of dTTP. The double strand cDNAs were subsequently purified, and single nucleotide adenine was added to the 3′ end. Then, multiple indexing adapters were ligated to both ends of the double strand cDNAs and enriched by PCR amplification. End preparation and three libraries including wild type, HFW1 and HFW3 were generated and sequenced on an Illumina HiSeq platform. To identify differentially expressed genes (DEGs), read counts were analyzed with edgeR v.3.5 package (McCarthy et al., 2012). The RNA sequencing data have been deposited in the NCBI Gene Expression Omnibus (GEO) database under the accession number GSE137468^1^ (token: gzqpigoonhgdjoh).

The up- or down-regulated DEGs having a Log_2_ fold change > 2 or <−2, respectively (*P* < 0.05), based on Student’s *t*-test were set as the threshold for significantly differential expression. By protein functional annotation^2^ and NCBI database search, we chose the genes with known or predicted function in *Fol* that were correlated with the fungal growth and virulence. For expression confirmation, 2 μg of total RNA was used for reverse transcription with a high-capacity cDNA reverse transcription kit (Applied Biosystems, CA, United States). qRT-PCR was carried out using 10 ng of cDNA as the template with SYBR Green PCR Master Mix (Thermo Fisher Scientific, Vilnius, Lithuania) in a StepOnePlus machine (Applied Biosystems, Foster City, CA, United States). The relative expression levels of *PMC1*, *YVC1*, *EGL7*, *SGE1*, *PSD2*, *HOG1*, *CNA1*, and *CNB1* genes were normalized to the *Actin* gene of *Fol*, by using the 2^–ΔΔ*Ct*^ method. Primer sequences for each gene are listed in Supplementary Table S1.

## Results

### Identification of Calcineurin in *F. oxysporum* f. sp. *lycopersici*

**FIGURE 1:**
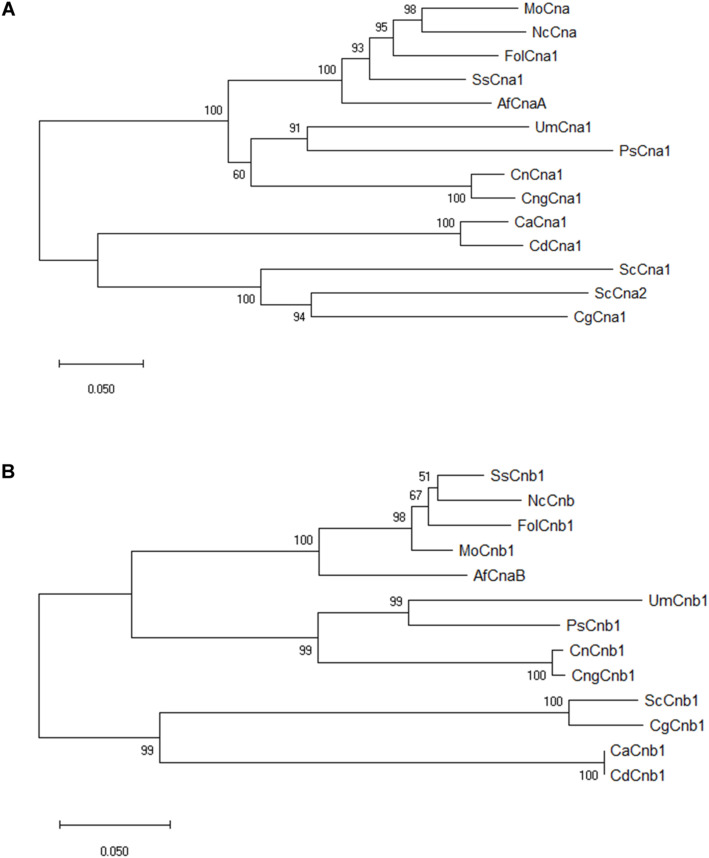
Phylogenetic analyses of *F. oxysporum* f. sp. *lycopersici* calcineurin orthologs in filamentous fungi and model organism. By comparing the selected orthologs of calcineurin catalytic Cna1 **(A)** and regulatory Cnb1 **(B)** subunits, phylogenic trees were constructed using MEGA software with the neighbor-joining method. The bootstrap confidence value was estimated by 1000 replicates. FolCna1 and FolCnb1 (*Fusarium oxysporum* f. sp. *lycopersici* 4287, XP_018243626 and XP_018234243), MoCna and MoCnb (*Magnaporthe oryzae* 70-15, XP_003711354 and XP_003709672), NcCna and NcCnb (*Neurospora crassa* OR74A, XP_961193 and CAA73345), SsCna1 and SsCnb1 (*Sclerotinia sclerotiorum* 1980 UF-70, XP_001597594 and XP_001598128), AfCnaA and AfCnaB (*Aspergillus fumigatus* Af293, XP_753703 and XP_747624), UmCna1 and UmCnb1 (*Ustilago maydis*, AAP48999 and EAK82139), PsCna1 and PsCnb1 (*Puccinia striiformis* f. sp. *tritici*, AFW98882 and AFW98883), ScCna1 and ScCna2 (*Saccharomyces cerevisiae*, AAA34465 and AAA34466), ScCnb1 (*Saccharomyces cerevisiae*, NP_012731), CnCna1 an CnCnb1 (*Cryptococcus neoformans* var. *neoformans*, AAD44336 and AAG13937), CngCna1 and CngCnb1 (*Cryptococcus neoformans* var. *gattii* WM276, XP_003196247 and XP_003196466), CaCna1 an CaCnb1 (*Candida albicans* SC5314, XP_718995 and XP_721917), CdCna1 and CdCnb1 (*Candida dubliniensis* CD36, XP_002416744 and XP_002420770), CgCna1 and CgCnb1 (*Candida glabrata*, XP_449251 and XP_448800).

The nucleotide sequence of calcineurin catalytic subunit A (XP_018243626) and regulatory subunit B (XP_018234243) were derived from the whole genome sequence of *Fusarium oxysporum* f. sp. *lycopersici* 4287 in the previous study (Ma et al., 2010). Both *CNA1* and *CNB1* genes having a single copy in the genome of *Fol* were verified by BLAST search^3^. The predicted open reading frame (ORF) of *CNA1* (XP_018243626) is 1,701 bp encoding a protein with 566 amino acids, which consists of several sub-domains including catalytic, calmodulin binding, calcineurin B subunit binding, and autoinhibitory domains (Supplementary Figure S1A). The predicted ORF of *CNB1* (XP_018234243) is 525 bp encoding a protein with 174 amino acids, which comprises four EF-hand calcium binding domains (Supplementary Figure S1B). Sequence comparison of *Fol* Cna1/Cnb1 amino acids and other previously reported orthologs in other phytopathogenic fungi and model organisms revealed that *Fol* Cna1 shares 86% identity with the calcineurin A subunit of *Magnaporthe grisea*, and 87% identity with *Sclerotinia sclerotiorum*. The *Fol* Cnb1 shares 93% identity with both calcineurin B subunit of *S. sclerotirum* and *M. grisea*. Phylogenetic analysis using neighbor-joining criteria performed with MEGA software^4^ showed that *Fol* Cna1 (Figure 1A) and Cnb1 (Figure 1B) cluster with other filamentous fungi but are distant from those of yeasts.

### Calcineurin Is Required for Phosphatase Activity in *F. oxysporum* f. sp. *lycopersici*

In order to understand whether Cna1 and Cnb1 are critical for calcineurin phosphatase activity in *Fol*, we generated two independent mutants of *cna1* and *cnb1*, respectively, by homologous recombination as described in Materials and Methods. The putative transformants were first screened by drug resistance (*Hyg*^*R*^) and green fluorescence (GFP) (Supplementary Figure S2A), and finally reconfirmed by PCR analysis (Supplementary Figure S2B). The *cna1* mutants (HFW1 and LHS2) and *cnb1* mutants (HFW3 and HFW4) were selected for further studies.

**FIGURE 2:**
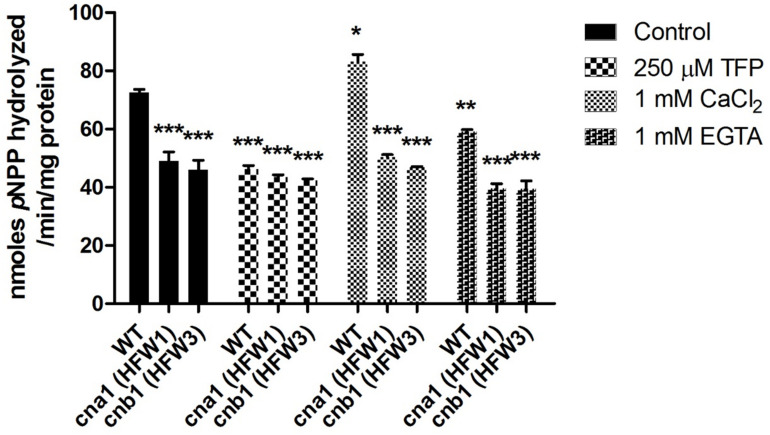
Calcineurin is required for phosphatase activity in *Fusarium oxysporum* f. sp. *lycopersici*. Phosphatase activity in the wild type and calcineurin mutants was determined after 72 h growth using *p*-nitrophenyl phosphate (pNPP) as the substrate. Ca^2+^ dependence of calcineurin activity was determined by adding 1mM CaCl_2_ or 1mM EGTA in the reaction. Inhibition of calcineurin activity was determined by adding 250 μM TFP in the reaction. Three technical replicates were performed, and results were analyzed by two-way ANOVA and followed by Bonferroni post-test. Error bars represent standard deviations. ^∗^*P* < 0.05; ^∗∗^*P* < 0.01; and ^∗∗∗^*P* < 0.001 were compared with the WT in the control group.

To examine whether the calcineurin activity was still exhibited in the mutants, the *p*-nitrophenyl phosphate (*p*NPP) was used as substrate for detection of phosphatase activity. Under normal treated condition, we found that phosphatase activity was reduced by approximately 40% in calcineurin mutants compared with the wild type (Figure 2). Concerning that *p*NPP might not be specific to the serine/threonine phosphatase, additional assays were carried out with or without calcineurin inhibitor (TFP, trifluoperazine) (Gupta et al., 1990), to verify whether the reduction of phosphatase activity in mutants was due to the inactivation of calcineurin. As shown in Figure 2, compared with the wild type under control treatment, enzyme activity had no difference between the wild type and calcineurin mutants in the presence of TFP, suggesting that the reduced phosphatase activity was a result of inactivation of calcineurin. To confirm the calcium dependence of *Fol* calcineurin activity, the Ca^2+^ and its chelator EGTA were supplemented into the reaction buffer, respectively. In the wild type, results showed that the Ca^2+^ and EGTA could elevate and decrease about 16.5 and 19.4% phosphatase activity, respectively, when compared with the untreated control (Figure 2), indicating the critical roles of Ca^2+^ in calcineurin activity of *Fol*.

### Calcineurin Governs Vegetative Growth of *F. oxysporum* f. sp. *lycopersici*

**FIGURE 3:**
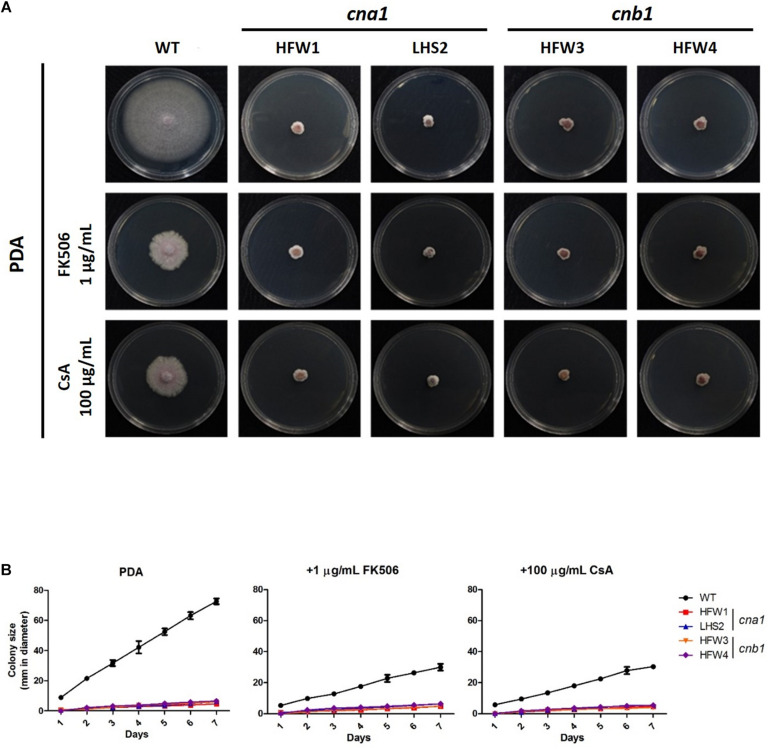
Genetic deletion and pharmacological inhibition of calcineurin cause growth defect in *Fusarium oxysporum* f. sp. *lycopersici*. **(A)** Pharmacological inhibition of calcineurin by calcineurin inhibitors partially mimics genetic deletion of calcineurin in causing growth defect of *F. oxysporum* f. sp. *lycopersici*. Vegetative growth of the wild type and calcineurin mutants in the absence or presence of a calcineurin inhibitor. All plates were incubated at 25°C for 1 week. **(B)** Growth kinetics of the wild type and calcineurin mutants in the presence or absence of a calcineurin inhibitor. Agar discs with the wild type or calcineurin mutants were put onto PDA, PDA + 1 μg/mL FK506, or PDA + 100 μg/mL cyclosporine A (CsA), and colony sizes of each strain were measured in diameter daily for 7 days. Error bars represent standard deviations from three technical replicates.

To investigate the roles of calcineurin in vegetative growth, the colony diameters of wild type and calcineurin mutants were examined daily after inoculation of mycelial agar disks. The wild type showed an extended and radiated mycelial growth on PDA medium. In contrast, the *cna1* mutants (HFW1 and LHS2) and *cnb1* mutants (HFW3 and HFW4) had compact, wrinkled and sunk colonies (Figure 3A). The average colony diameter of the wild type after 1-week measurement was 72.6 ± 1.0 mm, those of *cna1* mutants (HFW1 and LHS2) were 4.6 ± 0.6 and 6.0 ± 0.2 mm; and those of *cnb1* mutants (HFW3 and HFW4) were 6.0 ± 0.5 and 6.5 ± 0.2 mm (Figure 3B). The complementary strains (*cna1*:*CNA1* and *cnb1*:*CNB1*) showed colony morphology similar to the wild type (Supplementary Figure S4C). Although *cnb1*:*CNB1* grew slower, its growth can be restored as the wile type after slightly extended incubation time, while both *cna1* and *cnb1* mutants showed retarded growth regardless of extended incubation. These results demonstrated that reduced vegetative growth in *cna1* and *cnb1* mutants was due to the loss of calcineurin function.

In order to verify the vegetative growth defects caused by defective calcineurin activity, the calcineurin inhibitor FK506 or cyclosporine A was added to PDA medium. The average diameter of colonies was measured daily after inoculation. As shown in Figure 3, wild type showed a limited vegetative growth on PDA medium containing FK506 (1 μg/mL) or cyclosporine A (100 μg/mL), but was able to extend 29.6 ± 0.9 and 30.0 ± 0.8 mm after 1 week, respectively, whereas the growth of calcineurin mutants was retarded on medium with either inhibitor. In the presence of FK506, the average colony diameters of *cna1* mutants were 4.8 ± 0.4 mm (HFW1) and 6.3 ± 0.6mm (LHS2); and those of *cnb1* mutants were 4.8 ± 0.1 mm (HFW3) and 6.5 ± 0.5 mm (HFW4). In the presence of cyclosporin A, the average diameter of *cna1* and *cnb1* colonies was 4.6 ± 0.2 mm (HFW1), 5.5 ± 0.8 mm (LHS2), 4.1 ± 0.7 mm (HFW3), and 5.5 ± 0.5 mm (HFW4), respectively. These results indicate that pharmacological inhibition of calcineurin partially mimics genetic deletion of calcineurin in causing growth defects of *Fusarium oxysporum* f. sp. *lycopersici*.

### Calcineurin Controls Hyphal Development and Conidiation in *Fusarium oxysporum* f. sp. *lycopersici*

**FIGURE 4:**
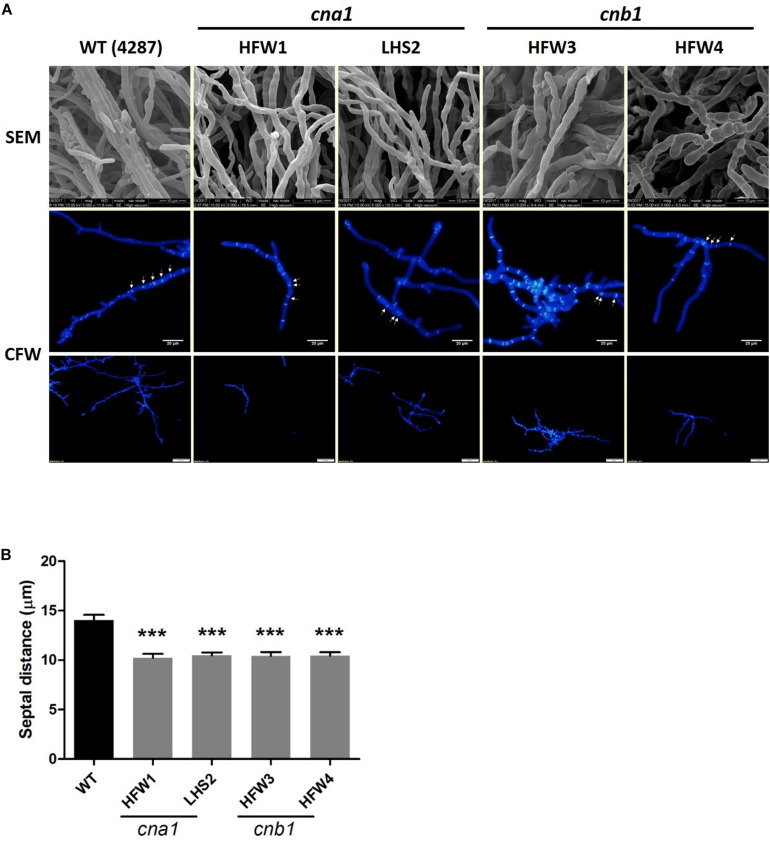
Irregular hyphae and abnormal distribution of septa in calcineurin mutants as revealed by SEM observation and CFW staining, respectively. **(A)** Hyphal development of 2-week-old wild type and calcineurin mutants was observed under a scanning electron microscope (SEM). Scale bars, 10 μm. Calcofluor white (CFW) staining of hyphae was used to determine the distance of the septa and distribution of hyphal compartment. Scale bars, 50 μm. **(B)** The average length of septal distance. Data were analyzed by one-way ANOVA. Asterisks indicate significant differences between wild type and calcineurin mutants (*P* < 0.001). Error bars represent standard errors of mean.

To further reveal the details of hyphal compartment in wild type and calcineurin mutants, strains were grown on PDA medium for 2 weeks followed by scanning electron microscope (SEM) analysis. Calcineurin mutants developed irregular, blunted and slightly swollen hyphae compared with the wild type (Figure 4A). Because calcineurin mutants developed abnormal hyphae and grew slower than the wild type, we hypothesized that calcineurin might be involved in hyphal development. To test this hypothesis, conidia suspension of wild type and calcineurin mutants were cultured overnight, and mycelia were harvested for calcofluor white (CFW) staining. Observation under a fluorescent microscope revealed that calcineurin mutants displayed an abnormal distribution of septa (Figure 4A). The average septal distance of wild type was 14.05 ± 4.47 μm, while those of calcineurin mutants were 10.22 ± 3.65 μm (HFW1), 10.48 ± 3.7 μm (LHS2), 10.42 ± 3.49 μm (HFW3), and 10.43 ± 3.2 μm (HFW4), respectively, resulting in a significant difference between the wild type and calcineurin mutants (Figure 4B).

**FIGURE 5:**
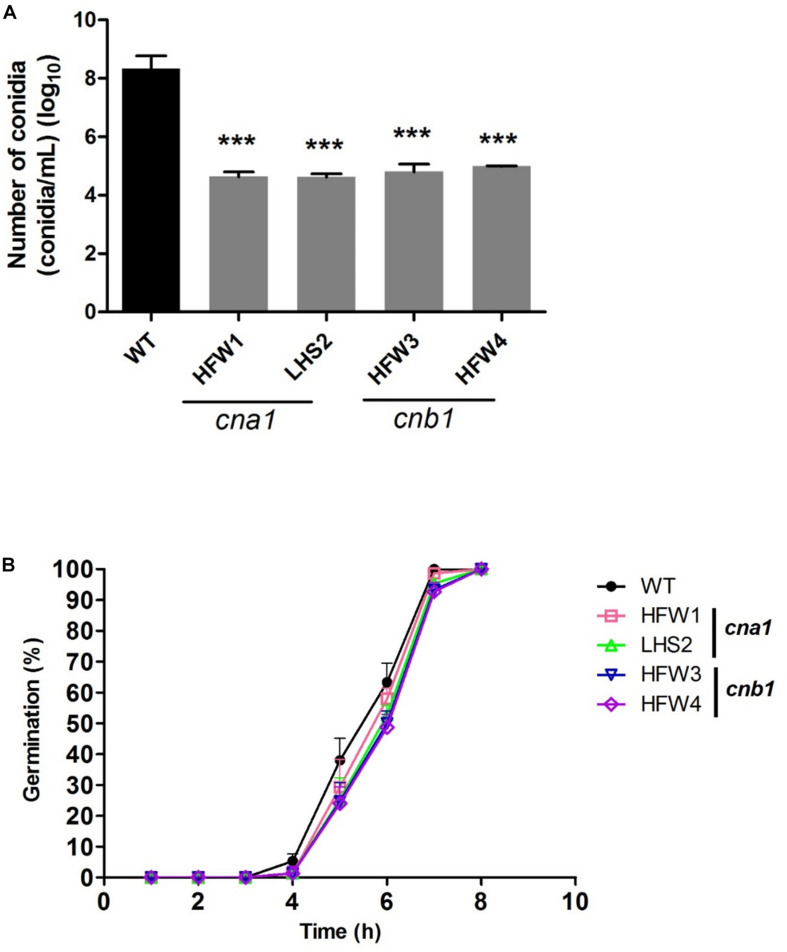
The conidia production and germination rate were reduced in calcineurin mutants. **(A)** Agar disks of wild type and calcineurin mutants were grown on PDA medium at 25°C for 2 weeks and the number of conidia was counted. Error bars represent standard deviations. Asterisks indicated significant differences (*P* < 0.001) between wild type and calcineurin mutants. **(B)** Conidia of the wild type and calcineurin mutants were suspended in PDB and adjusted to 10^5^ conidia/mL. The germination rate was calculated by randomly counting 50 conidia from each of 3 technical replicates over 8 h growth at 25°C.

We also monitored the production and germination rate of conidia in wild type and calcineurin mutants. The mycelia agar disks of strains were cultured on the PDA medium for 1 week, conidia were collected and resuspended in ddH_2_O for number calculation. The results showed that wild type produced more than 10^8^ conidia/mL, whereas *cna1* and *cnb1* mutants produced less than 10^5^ conidia/mL (Figure 5A), resulting in a larger than 1000-fold difference between the wild type and calcineurin mutants. To examine the rate of germination, conidia (10^5^conidia/mL) derived from the wild type and calcineurin mutants were cultured and germinated in PDB broth for 8 h. The germination rates were calculated at 1-h intervals. As shown in Figure 5B, *cna1* and *cnb1* mutants displayed a germination rate slightly slower than the wild type at 5 h (*P* < 0.01 in HFW1; *P* < 0.001 in LHS2, HFW3, and HFW4), while all strains reached nearly 100% germination rate at 8 h. These results demonstrated that calcineurin plays a vital role in hyphal growth and conidiation, but only slightly affects conidia germination in *Fol*.

### Calcineurin Mediates Chlamydospore Formation in *F. oxysporum* f. sp. *lycopersici*

**FIGURE 6:**
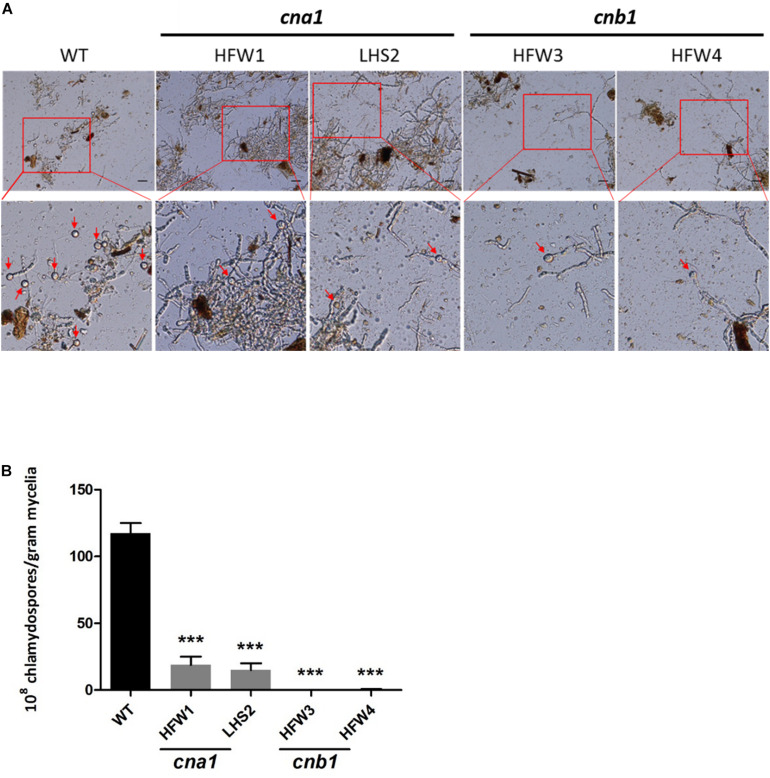
Chlamydospore production was impaired in calcineurin mutants. **(A)** Conidia from wild type and calcineurin mutants of *Fol* were inoculated into soil medium to induce chlamydospore formation and then collected as described in section “Materials and Methods.” Arrows indicate chlamydospores. The images in the rectangles of upper panel were enlarged in the lower panel. Scale bar, 50 μm. **(B)** The average number of chlamydospores produced by mycelia per gram from **(A)** was analyzed by one-way ANOVA and Bonferroni post-test. Error bars represent standard deviations. Asterisks indicate significant differences (*P* < 0.001) between wild type and calcineurin mutants. The data were derived from three technical replicates.

The role of calcineurin in fungal chlamydospore formation has not yet been reported. To examine this role, the number of chlamydospores were calculated in wild type and calcineurin mutants after incubation for 14 days in chlamydospore-inducing soil medium at 25°C. The number of chlamydospores were defined as the amount of chlamydospore produced per gram of dried mycelia as described in Materials and Methods. Our data showed that many chlamydospores were produced by wild type, but only a few were produced in calcineurin mutants (Figure 6A and Supplementary Figure S5). The chlamydospore production in wild type, *cna1* mutants (HFW1 and LHS2), and *cnb1* mutants (HFW3 and HFW4) was calculated as [117.5 ± 7.5] × 10^8^, [18.7 ± 6.2] × 10^8^, [15.0 ± 5.0] × 10^8^, [0.75 ± 0.0] × 10^8^, and [0.45 ± 0.35] × 10^8^ (chlamydospores/gram mycelia), respectively, resulting in a significant difference between the wild type and calcineurin mutants (Figure 6B).

### Calcineurin Is Required for Virulence of *F. oxysporum* f. sp. *lycopersici* on Tomato

**FIGURE 7:**
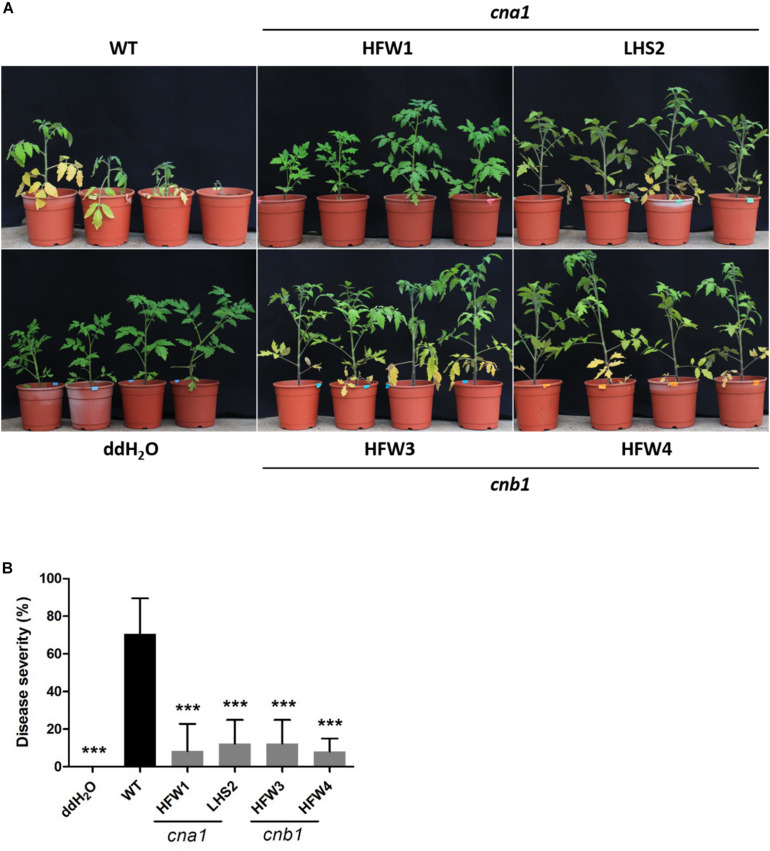
Calcineurin is critical for tomato infection in *F. oxysporum* f. sp. *lycopersici*. Tomato seedlings were inoculated by submerging roots in a suspension medium containing 5 × 10^6^ conidia/mL of wild type or calcineurin mutant for 30 min, followed by planting in horticultural substrates, and maintaining in the greenhouse at 28°C. **(A)** Representative plant phenotypes after 21 days of inoculation. **(B)** Disease severity was evaluated after 21 days of inoculation. Error bars represented standard deviations. Asterisks indicated significant differences between wild type and calcineurin mutants or mock treatment (*P* < 0.001).

To evaluate the virulence of *Fol* wild type and calcineurin mutants, we conducted a tomato infection assay by the root-dipping inoculation method. Two-week-old tomato seedlings were each inoculated with wild type or a calcineurin mutant, and then grown for 21 days in a green house. The wild type infected tomato plants showed typical Fusarium wilt symptoms, such as yellowing leaves and browning vascular tissues, while only a few leaves displayed yellowing symptoms in calcineurin mutant inoculated seedlings (Figure 7A). All plants wilted and died at 30 days post inoculation (dpi) after wild type infection, whereas plants inoculated with calcineurin mutants revealed a dramatic reduction in disease symptoms and no death was observed at 30 dpi. After 21 dpi, the disease severity of infection with wild type was 100%, and 30, 10, 0 and 0% for the HFW1, LHS2, HFW3 and HFW4, respectively (Figure 7B). Meanwhile, histological observation of tomato infected with wild type or calcineurin mutant was conducted. Cross-section the stem 1 cm above the ground after 21 dpi, as shown in Supplementary Figure S6, the mycelium of *Fol* wild type could colonize pith, xylem, phloem, and cortex cells of tomato seedlings (Supplementary Figure S6B). Whereas no hyphae can be found in the vascular tissues or cortex cells when tomato seedlings inoculated with *cna1* or *cnb1* mutants (Supplementary Figures S6C,D). Based on these results, we suggested that calcineurin mutants could invade tomato root but unable to colonize in vascular tissue, indicating ting that calcineurin might be contributed to virulence of *Fol* on tomato host.

### RNA Sequencing Analysis of Calcineurin-Mediated Genes in *F. oxysporum* f. sp. *lycopersici*

**FIGURE 8:**
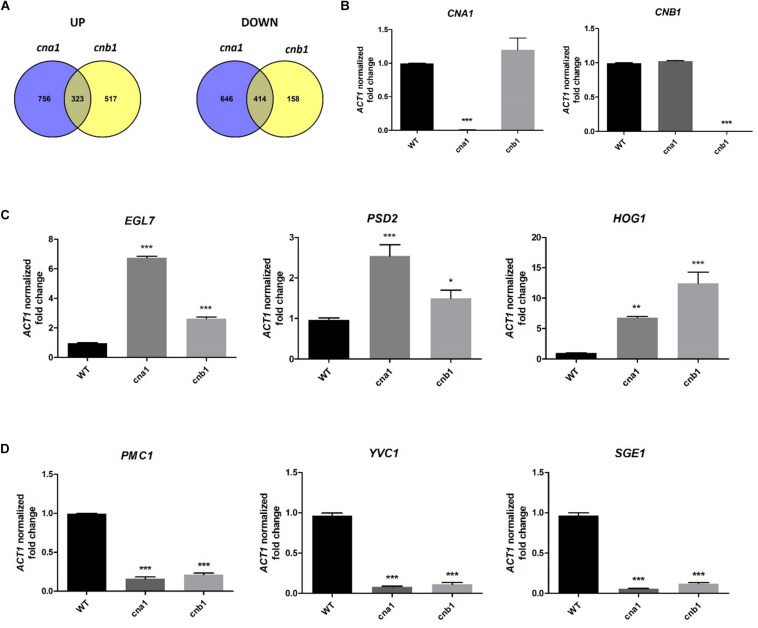
Genes regulated by calcineurin in *Fusarium oxysporum* f. sp. *lycopersici*. The 10^6^ conidia/mL of wild type and calcineurin mutants were inoculated in PDB media, and mycelia were collected for RNA extraction after incubation at 25°C for 14 h. **(A)** Venn diagram illustrating the number of genes expressed differentially between the wild type and *cna1* (HFW1) or *cnb1* mutant (HFW3) (Log_2_FC > 2 or < –2, and *P* < 0.05). A total of 323 genes were upregulated, and 414 genes were downregulated in both *cna1* and *cnb1* mutants as compared with the wild type. **(B)** The relative expression levels of *CNA1* or *CNB1* genes in wild type, *cna1* or *cnb1* mutant by qRT-PCR analysis. The expression profiles of three upregulated (*EGL7*, *PSD2*, *HOG1*) **(C)** and three downregulated genes (*PMC1*, *YVC1*, *SGE1*) **(D)** compared to the wild type were confirmed by qRT-PCR. Error bars represent standard deviation, and results are representative of three technical replicates of one out of three biological replicates, which showed similar results. Asterisks indicated significant differences compared to wild type according to one-way ANOVA and Dunnett’s post-test. *P < 0.05, **P < 0.01, and ***P < 0.001.

To further understand the pleiotropic phenotypes of calcineurin mutants in *Fol*, we identified calcineurin-mediated genes via RNA sequencing analysis. Strains were grown in PDB at 25°C for 14 h and total RNA was extracted for RNA sequencing analysis. By pairwise analysis, a total of 2139 genes were differentially expressed in wild type and *cna1* mutant (HFW1) based on the selection criteria of four-fold change in expression (Log_2_ fold change > 2 or < -2, and *P* < 0.05 based on Student’s *t*-test). Among these, 1079 genes were upregulated and 1060 genes were downregulated in *cna1* mutant (Figure 8A). From the comparison of wild type against *cnb1* mutant (HFW3), 840 genes were upregulated while 572 genes were downregulated in *cnb1* mutant (Figure 8A).

Based on the Venn diagram analysis with selection criterion of fourfold change in expression, we identified 737 genes that were differentially expressed coordinately in both *cna1* and *cnb1* mutants, among which 323 (20% overlapping, 323/1596) and 414 (34% overlapping, 414/1218) genes were up- and down-regulated, respectively (Figure 8A). Moreover, gene ontology (GO) enrichment analysis was performed to better understand the function of those differentially expressed genes (DEGs) that coordinately regulated in both *cna1* and *cnb1* mutants. The up- and down-regulated genes are functionally correlated with the cellular component, molecular function and biological process, such as those related to cell membrane (GO:0016020, GO:0005886), cell periphery (GO:0071944), and transmembrane transporter (GO:0022891, GO:0022892) (Supplementary Figure S7).

Previous reports demonstrated that calcineurin is involved in ion homeostasis, hyphal growth, cell wall integrity and virulence in filamentous fungi (Juvvadi et al., 2014). We, therefore, screened from those transcripts that overlapped in both *cna1* and *cnb1* mutants that were associated with the above biological functions. As shown in Table 2 (only showing genes functionally annotated in *Fol*), out of 737 transcripts, 7 were found to be related to cell wall integrity. Among which 6 genes were upregulated and found to encode endo-β-1,4-glucanase (*FolEGLD*), endoglucanase (*FolEGL4*, *FolEGL7*), chitin synthase (*FolCHSD)*, phosphatidylserine decarboxylase (*FolPSD2*), and protein kinase (*FolHOG1*); in addition, one gene encoding chitinase (*FolCHS1*) was found to be downregulated. As well as cell wall synthesis-related genes, those involved in the transportation of ions and small molecules were also found to be regulated. Three genes were downregulated, P-type Ca^2+^ ATPase (*FolPMC1*), Mg^2+^ translocating P-type ATPase (*FolMGTB*), and Ca^2+^ channel (*FolYVC1*); and one gene encoding Cu^2+^ exporting ATPase (*FolCCC2*) was upregulated (Table 2). Interestingly, we found a nuclear protein, SIX gene expression 1 (Sge1), a gene essential for pathogenicity in *Fol*, was downregulated in both calcineurin mutants.

**TABLE 2:** Calcineurin-regulated genes are related to cell wall integrity, cation homeostasis, and transcriptional regulation.

**Gene name**	**Description**	**Transcript ID**	***cna1*/WT Log_2_FC^a^**	***cnb1*/WT Log_2_FC^a^**
**Cell wall integrity**				
*EGL4*	Endoglucanase	FOXG_04120	2.85	2.54
*EGL7*	Endoglucanase	FOXG_05654	7.99	6.32
*EGLD*	Endo-β-1,4-glucanase	FOXG_09647	6.49	3.95
*CHSD*	Chitin synthase	FOXG_04179	2.46	2.49
*CHI1*	Chitinase	FOXG_10748	–3.90	–2.99
*PSD2*	Phosphatidylserine decarboxylase	FOXG_16677	3.65	2.53
*HOG1*	CMGC protein kinase	FOXG_13701	2.72	8.72
**Cation homeostasis**				
*CCC2*	Cu^2+^ exporting ATPase	FOXG_12226	3.94	4.44
*MGTB*	Mg^2+^ translocating P-type ATPase	FOXG_02777	–9.25	–4.30
*PMC1*	Ca^2+^ transporting ATPase	FOXG_06211	–3.34	–2.80
*YVC1*	Ca^2+^ channel	FOXG_13541	–8.15	–6.22
**Transcriptional regulation**				
*SGE1*	Six gene expression protein 1	FOXG_10510	–3.96	–2.68

*Gene name and description were identified using the UniProtKB and NCBI database. ^*a*^Log_2_FC, Log_2_ fold change.*

To validate the reliability of the candidate gene expression data obtained from RNA sequencing results, the transcription levels of three upregulated genes (*EGL7*, *PSD2*, *HOG1*) and three downregulated genes (*PMC1*, *YVC1*, *SGE1*) were determined by quantitative real-time RT-PCR (qRT-PCR). As shown in Figure 8B, qRT-PCR results confirmed that *CNA1* or *CNB1* transcription was abolished in *cna1* or *cnb1* mutant, respectively (Figure 8B). Meanwhile, the relative expression levels of *EGL7*, *PSD2*, and *HOG1* were increased in both *cna1* and *cnb1* mutants (Figure 8C), while the expression of *PMC1*, *YVC1*, and *SGE1* were reduced in both *cna1* and *cnb1* mutants as compared with the wild type (Figure 8D). These data were consistent with RNA sequencing results.

## Discussion

In this study, the biological function of two calcineurin genes, *FolCNA1* and *FolCNB1*, from the tomato wilt fungus *F. oxysporum* f. sp. *lycopersici* was characterized. Calcineurin is globally conserved and found in all eukaryotes (Chen et al., 2010; Park et al., 2019). By phylogenetic tree analysis, our results showed that FolCna1 and FolCnb1 were closely related to calcineurin orthologs from other Ascomycete fungi (Figure 1), indicating that functional similarity of calcineurin might be present among the Ascomycetous fungi.

*Fol* Calcineurin mutants showed severe defects in vegetative and hyphal growth, and a dramatic decrease in virulence. The colony of calcineurin mutants grown on the PDA media were compact and stunted and the radial growth was severely restricted, whereas the wild type displayed longer and dispersed hyphae (Figure 3). These features were similar to the calcineurin A mutant of *A. fumigatus* and *Botrytis cinerea* reported in previous studies, which had a tiny, dense, and extremely blunted colony (Steinbach et al., 2006; Juvvadi et al., 2008; Harren et al., 2012). Moreover, blunted hyphae and shorter hyphal compartments could be found in *Fol* calcineurin mutants under SEM observation and CFW staining (Figure 4). These morphological changes indicate that cell wall integrity might be altered in *Fol* calcineurin mutants.

*Fol* calcineurin mutants had a partial reduction in phosphatase activity as compared to the wild type (Figure 2), suggesting that other components or heterodimer partner might contribute to the remaining phosphatase activity. It will be interesting to test whether deletion of both *CNA1* and *CNB1* genes in *Fol* will abolish phosphatase activity. Meanwhile, a pull-down assay using either *Fol* calcineurin subunit as bait might identify a component interacting with calcineurin that has phosphatase activity or calcineurin associated function (Rodríguez et al., 2009; Park et al., 2016).

The soil inhabitant phytopathogenic fungus *Fol* could survive in plant residues or soil as mycelia or spores during the absence of a host (Agrios, 2005). In the field, *Fol* mainly survives as a type of chlamydospore, a thick-walled dormant propagule. Extensive studies have shown that chlamydospores play important role in maintaining the *Fusarium* wilt disease and causing more severe disease symptoms than those of macroconidia or microconidia (Gordon, 2017; Srinivas et al., 2019). In our findings, *cna1* and *cnb1* mutants reduced conidia production (Figure 5) and impaired chlamydospore formation (Figure 6). Moreover, many of the chlamydospores formed in *cna1* or *cnb1* mutants have oval-like structure with enlarged suspensor cell instead of typical round structure, indicating that these chlamydospores might be immature or malformed (Supplementary Figure S5) and consequently reduced the disease incidence of tomato Fusarium wilt and affected the life cycle of *Fol*. However, the conidia germination rates were only slightly altered in *cna1* and *cnb1* mutants compared with the wild type, indicating that calcineurin plays an important role in the production of conidia and chlamydospore. To the best of our knowledge, calcineurin has not previously been found to affect the formation of chlamydospore in fungi, our findings therefore define a new role for calcineurin in *Fol*.

Several studies have shown that calcineurin is indispensable for pathogenicity in phytopathogenic fungi. A calcineurin gene *MCNA* silenced by RNA interference in rice blast fungus *Magnaporthe. oryzae* resulted in a drastic reduction in mycelial growth, conidiation, appressorium formation, and pathogenicity (Choi et al., 2009). The expression of antisense *CNA1* in *S. sclerotiorum* led to the inhibition of hyphal growth, decreased sclerotia production, and attenuated pathogenicity (Harel et al., 2006). The calcineurin A mutant in gray mold fungus *B. cinerea* displayed severe defects in hyphae growth which caused a failure to penetrate the bean leaves (Harren et al., 2012). Our studies showed that calcineurin is critical for hyphal growth, conidia production, septal distribution and virulence in *Fol*. These results revealed that calcineurin plays a vital role in virulence of phytopathogenic fungi.

Due to the importance of calcineurin in fungal growth and virulence, it serves as an ideal antifungal drug. The calcineurin inhibitor such as FK506 or cyclosporin A can bind to FKBP12 or cyclophilin, respectively, and formed a complex with calcineurin and subsequently blocked the phosphatase activity. However, because calcineurin was a conserved phosphatase in eukaryotes, calcineurin inhibitor would also cause immune suppression in mammals. A previous study in our lab revealed that a non-conserved motif within fungal FKBP12 by crystal structure analysis and developed an FK506 analog (APX879), which could attenuate immune suppression in mammalian but enhance antifungal activity in animal therapeutic models (Juvvadi et al., 2019). Moreover, a Serine-Proline Rich Region (SPRR) was found to be a unique domain in filamentous fungal calcineurin, and mutation of the phosphorylated serine residues in the SPRR could block the phosphorylation of CnaA in *A*. *fumigatus* (Juvvadi et al., 2013), providing a potential fungal-specific drug target of calcineurin inhibitor.

According to the transcriptome analysis, a few genes related to fungal cell wall integrity were found to be regulated in *Fol* calcineurin mutants. The cell wall degrading enzymes (*EGLD*, *EGL4*, *EGL7*) and chitin synthase (*CHSD*) were abundantly expressed in both *cna1* and *cnb1* mutants (Table 2). In *A. fumigatus*, it has been shown that β-1,3-glucanase and chitinase plays an important role in hyphal extension by cleavage of the long chain of β-1,3-glucan and broke down the glycosidic bonds of chitin, respectively, both resulting in cell wall remodeling (Hartl et al., 2011; Langner and Gohre, 2016). In addition, the expression of putative *HOG1* ortholog, one of MAPK family members, was coordinately regulated in both *Fol cna1* and *cnb1* mutants. Previous research also showed that the Hog signaling pathways were involved in cell wall integrity of *C. neoformans* (Bahn and Jung, 2013), indicating that calcineurin might affect cell wall integrity via regulating Hog signaling in *Fol*. Our study revealed that the colony growth of calcineurin mutants grown on PDA medium containing CFW was similar to those grown on PDA medium only, and the protoplast formation efficiency had no significant difference between wild type and calcineurin mutants (Supplementary Figure S8). As a result, we supposed that the fungal cell wall interference agents (CFW and lysing enzyme) may not have significant effect on *Fol* calcineurin mutants. Collectively, these results suggested that the biosynthesis of the fungal cell wall might be disturbed in *Fol* calcineurin mutants, thereby resulting in abnormal hyphal development. We therefore presume that calcineurin might be required for septa formation and proper deposition.

The calcium signaling pathways are critical for virulence, infection, or survival in many pathogenic fungi (Liu et al., 2015). The vacuolar calcium channel *YvcA* (*YVC1* ortholog) in *A. fumigatus* was responsible for transferring calcium from the cytoplasm to the vacuoles, and the null mutation of *YvcA* displayed attenuated virulence in the murine model (de Castro et al., 2014). In addition, the P-type ATPase, encoded by *PMC1* has been identified as a downstream regulated gene of calcineurin in eukaryotic cells, which is involved in calcium sequestration and regulation. Previous studies in *Candida* spp. demonstrated that the expression of *PMC1* was regulated by calcineurin (Sanglard et al., 2003; Chen et al., 2011), and was required for pathogenesis in *C. albicans* (Luna-Tapia et al., 2019). The *pmcA* (*PMC1* ortholog) mutant in *A. fumigatus* was found to be avirulent (Dinamarco et al., 2012). Our results demonstrated that *Fol cna1* and *cnb1* mutants showed reduced virulence in tomato plants (Figure 7) and downregulated expression of *FolPMC1* and *FolYVC1* in both calcineurin mutants (Table 2). Additionally, according to our transcriptomic analysis, SIX gene expression 1 (*SGE1*), a transcription factor in *Fol*, was downregulated in both *cna1* and *cnb1* mutants (Table 2). Deletion of *FolSGE1* resulted in loss of virulence and inhibited the expression of the effector proteins (secreted in xylem proteins, SIX) during colonization in tomato plant (Lievens et al., 2009; Michielse et al., 2009b). Together, these studies indicate that *Fol* calcineurin control of virulence is potentially, at least in part, via regulating downstream genes such as *YVC1*, *PMC1*, or *SGE1*. Further experiments to overexpress *FolYVC1*, *FolPMC1*, or *FolSGE1* in *Fol* calcineurin mutant might provide further clarification about this influence.

## Conclusion

The deletion of *CNA1* and *CNB1* in *Fol* resulted in impaired vegetative growth, irregular hyphal development, and the reduction of conidia production and chlamydospore formation. *Fol* calcineurin mutants also contribute to attenuated virulence in tomato plants. The transcriptome analysis revealed the expressions of cell wall integrity and virulence-related genes were regulated by calcineurin. Taken together, our findings demonstrate that calcineurin signaling plays vital roles in conidiation, chlamydospore formation and virulence of *Fol*.

## Data Availability Statement

The RNA sequencing data have been deposited in the NCBI Gene Expression Omnibus (GEO) database under the accession number GSE137468 (https://www.ncbi.nlm.nih.gov/geo/query/acc.cgi?acc=GSE137468, token: gzqpigoonhgdjoh).

## Author Contributions

Y-HH, L-HH, and Y-HL designed the experiments. H-FW and L-HH generated the calcineurin mutants. Y-HH conducted the experiments and wrote the manuscript. Y-LC supervised, designed the experiments, analyzed and interpreted the data, and wrote the manuscript. All the authors read and approved the manuscript.

## Conflict of Interest

The authors declare that the research was conducted in the absence of any commercial or financial relationships that could be construed as a potential conflict of interest.

## Footnotes

1 https://www.ncbi.nlm.nih.gov/geo/query/acc.cgi?acc=GSE137468

2 https://www.uniprot.org/uploadlists/

3 https://www.ncbi.nlm.nih.gov

4 https://www.megasoftware.net/

## Supplementary Material

Supplementary Figure 1 Schematic representation of calcineurin domains in *F. oxysporum* f. sp. *lycopersici* 4287. **(A)** The catalytic subunit *CNA1* encodes a protein of 566 amino acids, which consists of a catalytic domain, a Cnb binding helix (BBH), a calmodulin-binding domain (CaMBD), and an autoinhibitory domain (AID). **(B)** The regulatory subunit Cnb1 encodes a protein of 174 amino acids, comprising of four EF-hand Ca^2+^-binding domains.

Supplementary Figure 2 Green fluorescence protein observation and PCR analysis of *F. oxysporum* f. sp. *lycopersici* wild type and calcineurin mutants. Green fluorescence protein (GFP) fluorescence **(A)** and PCR analysis **(B)** were used to confirm the correct disruption of *FolCNA1* and *FolCNB1*. Scale bars, 50 μm. Primers used to confirm wild type and calcineurin mutants are marked with numbers 1 to 6.

Supplementary Figure 3 Schematic diagram of *FolCNA1* and *FolCNB1* disruption construct. A diagram showing targeted gene disruption in *Fol* non-coding region (NCR) flanking 5′ and 3′ ends of the *FolCNA1* **(A)** or *FolCNB1* **(B)** gene was fused with the hygromycin resistance cassette under the control of P*gpdA* promoter and T*trpC* terminator by fusion PCR, and further transformed into the protoplast of the wild type (4287) to carry out homologous recombination. Arrows represent primers used to amplify specific genes or to verify whether successful for targeted DNA replacement.

Supplementary Figure 4 The growth defects caused by the genetic deletion of *CNA1* and *CNB1* were restored in the complemented strains. **(A)** Schematic diagram of *cna1* and *cnb1* complementation construct. A diagram showing *FolCNA1* or *FolCNB1* open reading frame (ORF) fused with the bleomycin resistance cassette by fusion PCR, and further transformed into the protoplast of the *cna1* or *cnb1* mutant, respectively, to carry out homologous recombination. Arrows represent primers used to amplify specific genes or to verify whether successful for targeted DNA replacement. **(B)** PCR analysis was used to confirm the complementary strains (*cna1*:*CNA1* and *cnb1*:*CNB1*). Primers were listed in Supplementary Table S1. **(C)** Radial growth of the wild type, calcineurin mutants, and complementary strains. All plates were incubated at 25°C for 1 week. **(D)** Growth kinetics of the wild type and complementary strains. Colony sizes of each strain were measured in diameter daily for 7 days. Error bars represent standard deviations of three technical replicates.

Supplementary Figure 5 Calcofluor white (CFW) and Nile red (NR) staining of *Fol* wild type and calcineurin mutants. Conidia from wild type and calcineurin mutants were inoculated into soil extract to induce chlamydospore formation and then collected as described in section “Materials and Methods.” Arrows indicate the chlamydospores. Scale bar, 20 μm.

Supplementary Figure 6 Histological observation in stem cross sections of tomato seedlings inoculated with the *Fol* wild type or calcineurin mutant. The 2-week-old tomato seedlings were inoculated with a suspension of 5 × 10^6^ conidia/mL of *Fol* wild type or calcineurin mutant by submerging roots, and then transplanting into green house for 21 days. Cross section conducted at the stems 1 cm above the ground were examined and photographed. Tomato seedling inoculated with dH_2_O. Scale bar, 200 μm **(A)**, *Fol* wild type. Scale bar, 200 μm **(B)**, *cna1* mutant. Scale bar, 200 μm (left) and 50 μm (right) **(C)**, or *cnb1* mutant. Scale bar, 200 μm **(D)**. Arrows indicate the infected hyphae constitute a cross network between cortex cells and cell surface, while inoculated with *Fol* wild type **(B)**. Whereas no obvious hyphae can be found on the cortex cells or cell surface, while seedlings inoculated with dH_2_O or calcineurin mutants **(A,C,D)**. (pi: pith; co: cortex; xy: xylem; ph: phloem).

Supplementary Figure 7 Gene ontology enrichment analysis of the DEGs that coordinately regulated in both *cna1* and *cnb1* mutants. Genes are functional correlated with the Cellular component **(A)**, Molecular function **(B),** and **(C)** Biological process.

Supplementary Figure 8 The cell wall integrity analysis of *Fol* wild type and calcineurin mutants. **(A)** The cell wall interference agent calcofluor white (CFW) can slightly reduce the colony growth of calcineurin mutants compared with the wild type. Vegetative growth of the wild type and calcineurin mutants was observed in the absence or presence of CFW. All plates were incubated at 25°C for 3 days and photographed. **(B)** The protoplast formation efficiency has no significant difference between the wild type and calcineurin mutants. The 10^8^ conidia/mL of the wild type and calcineurin mutants were inoculated in PDB media and incubated at 25°C for 16 h, then mycelia were collected and protoplasted with 10 mg/mL lysing enzyme. The protoplasts were counted using a hemocytometer.

Supplementary Table 1 Primers used in this study.
