# Supplementary material for: Calcineurin Regulates Conidiation, Chlamydospore Formation and Virulence in Fusarium oxysporum f. sp. lycopersici
Source: Front Microbiol. 2020 Oct 22;11:539702. doi: 10.3389/fmicb.2020.539702 (PMC7641966; doi:10.3389/fmicb.2020.539702)
Supplement: Supplementary Figure 1 — Schematic representation of calcineurin domains in F. oxysporum f. sp. lycopersici 4287. (A) The catalytic subunit CNA1 encodes a protein of 566 amino acids, which consists of a catalytic domain, a Cnb binding helix (BBH), a calmodulin-binding domain (CaMBD), and an autoinhibitory domain (AID). (B) The regulatory subunit Cnb1 encodes a protein of 174 amino acids, comprising of four EF-hand Ca2+-binding domains. [file Data_Sheet_1.PDF]

**Supporting Information**

**Table S1. Primers used in this study.**

| **Primer** | **Use** | **Sequence (5' to 3')** |
|---|---|---|
| JC753 | F-5'NCR of *CNA1* | AAATTAATTAAAAGGTATTACATTGAATATGA |
| JC756 | R-3'NCR of *CNA1* | AAATCTAGATTTCAGGTCTCGTGAATGGA |
| JC766 | F-*hyg*$^R$ from pPK2-hphgfp | GAGCTCGGTACCCGGGGATCT |
| JC767 | R-*hyg*$^R$ from pPK2-hphgfp | AAGAAGGATTACCTCTAAACA |
| JC768 | R-5'NCR of *CNA1* | GATCCCCGGGTACCGAGCTCGCCCAATTAGAAGATAGCAG |
| JC769 | F-3'NCR of *CNA1* | GCGAATTGGAGCTCCACCGCGGCTTCCTTACCCTTGATACCC |
| JC772 | F-ORF of *CNA1* | GTACCCGAGATCGATTTTACT |
| JC773 | R- ORF of *CNA1* | TAGTCACAGGTTTGTGGCCAG |
| JC836 | F- *Bleo*$^R$ cassette | GAGCTCGGTACCCGGGGAT |
| JC837 | R-3'NCR of *CNA1* with 20 nt *Bleo*$^R$ cassette | TGTTTAGAGGTAATCCTTCTTTTCTTTGGATACTGGCGGT |
| JC838 | R-ORF of *CNA1* with 20 nt *Bleo*$^R$ cassette | ATCCCCGGGTACCGAGCTCGGGTTGCAGCGTTTTACACCA |
| JC839 | F-5'NCR of *CNB1* | AGGTACCTATGACGCTTGGA |
| JC840 | R-5'NCR of *CNB1* | AGATCCCCGGGTACCGAGCTCCGCAAAGGGGCAAGTGCGC |
| JC841 | F-3'NCR of *CNB1* | TGTTTAGAGGTAATCCTTCTTTTTCTTGATTACTTACCAA |
| JC842 | R-3'NCR of *CNB1* | CTGAGCGAGGGCATATTCCG |
| JC843 | F-ORF of *CNB1* | TCGCCACAACCTAACCCAAC |
| JC844 | R- ORF of *CNB1* | CATCACCCCTCCCCCAACCG |
| JC1405 | F- *Bleo*$^R$ cassette | AAGTTGACCAGTGCCGTTCC |
| JC1404 | R- *Bleo*$^R$ cassette | CACGAAGTGCACGCAGTTG |
| JC1452 | F-*FoAct* for qPCR | ATCCACGTCACCACTTTCAA |
| JC1453 | R-*FoAct* for qPCR | TGCTTGGAGATCCACATTTG |
| JC1952 | F-*FoPMC1* for qPCR | TAAACAACATCGCCCCATGC |
| JC1953 | R-*FoPMC1* for qPCR | GCGATGAGCGACAGCCTAGA |
| JC1954 | F-*FoYVC1* for qPCR | AAACCCCCACCCAAAATTCC |
| JC1955 | R-*FoYVC1* for qPCR | GCCACACTGCACTGCTCCTT |
| JC1956 | F-*FoSGE1* for qPCR | AGCCGAGGAAAATGGCAGAG |
| JC1957 | R-*FoSGE1* for qPCR | CTCTGGCCTTGCGGCTATCT |
| JC1958 | F-*FoEGL7* for qPCR | CATTGCCTGCCACAAGAAGG |
| JC1959 | R-*FoEGL7* for qPCR | CGCACTTGGCAACGTAGGTC |
| JC1960 | F-*FoPSD2* for qPCR | TCCAGCATCCAAGGAGCAAA |
| JC1961 | R-*FoPSD2* for qPCR | CTTGAATCGGCGACAACGAC |
| JC1962 | F-*FoHOG1* for qPCR | GGACCTGGGAGAAACCATCG |
| JC1963 | R-*FoHOG1* for qPCR | CGCGCAGAAGTTGTCTTGCT |
| JC1964 | F-*FoCNA1* for qPCR | TTTCCGCGAGCCTCCTACTC |
| JC1965 | R-*FoCNA1* for qPCR | TGAGCATCCTCGGACATGGT |
| JC1966 | F-*FoCNB1* for qPCR | CGGTGACGTCGATTTCCAAG |

| | | |
|---|---|---|
| JC1967 | R-*FoCNB1* for qPCR | TTGCTGCCGACCATCATCTT |

A

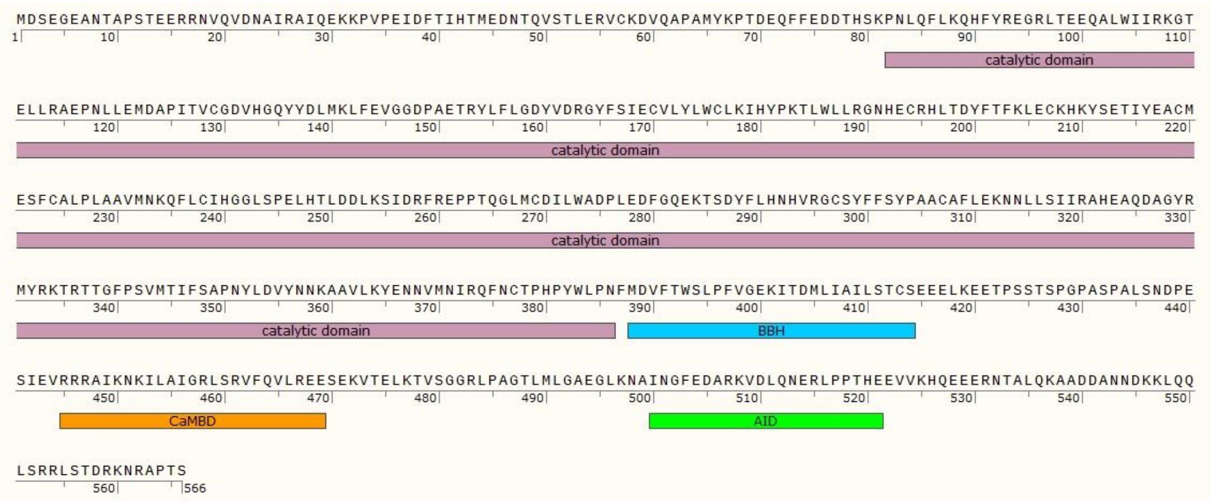


B

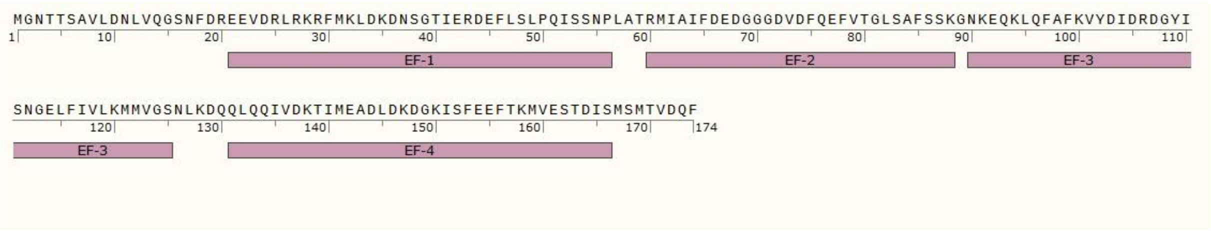


**Figure S1. Schematic representation of calcineurin domains in *F. oxysporum* f. sp. *lycopersici* 4287. (A)** The catalytic subunit Cna1 includes 566 amino acids, comprising of a catalytic domain, a Cnb binding helix (BBH), a calmodulin-binding domain (CaMBD), and a autoinhibitory domain (AID). **(B)** The regulatory subunit Cnb1 includes 174 amino acids, comprising of four EF-hand $Ca^{2+}$-binding domains.

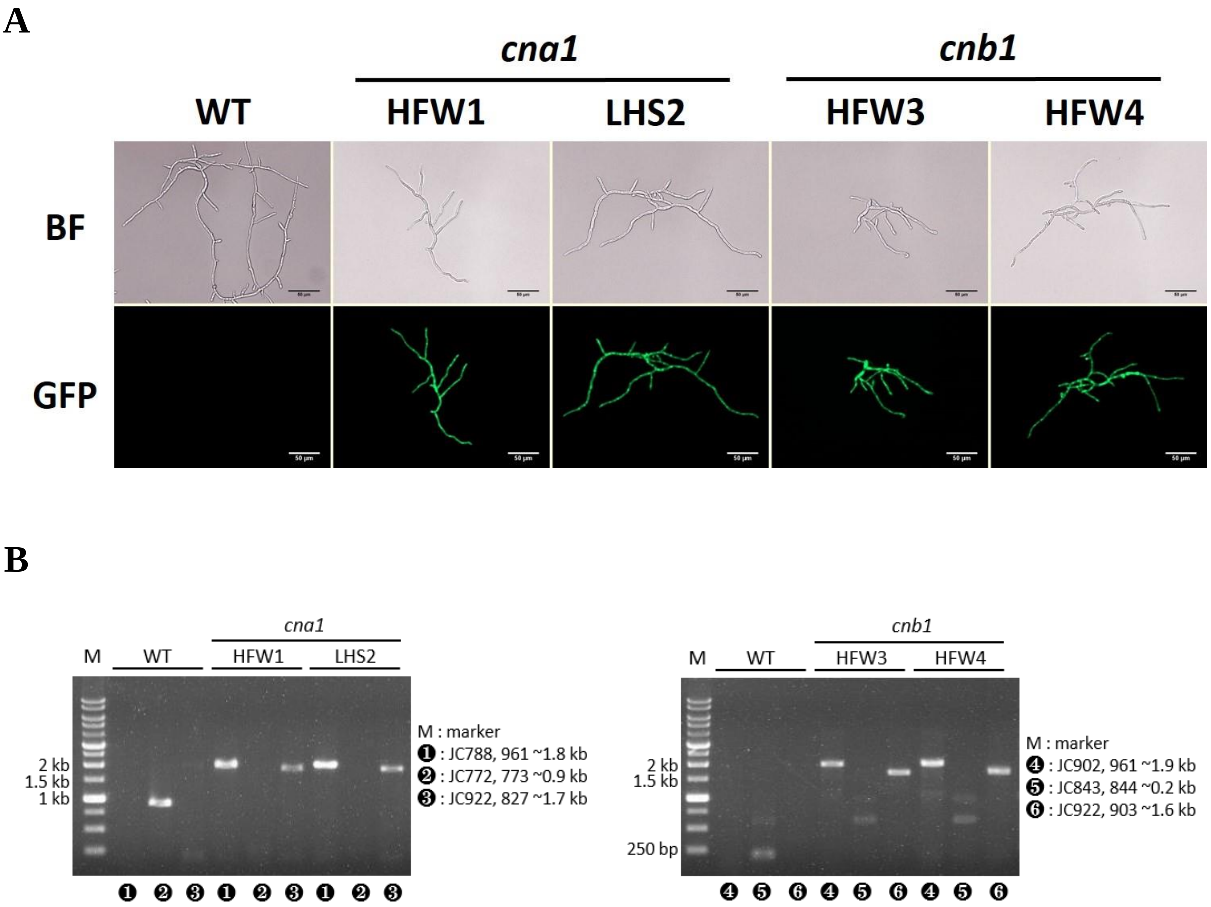


**Figure S2. Green fluorescence protein observation and PCR analysis of *Fol* wild type and calcineurin mutants.** Green fluorescence protein (GFP) fluorescence **(A)** and PCR analysis **(B)** were used to confirm the correct disruption of *FolCNA1* and *FolCNB1*. Scale bars, 50 µm. Primers used to confirm wild type and calcineurin mutants are marked with numbers 1 to 6.

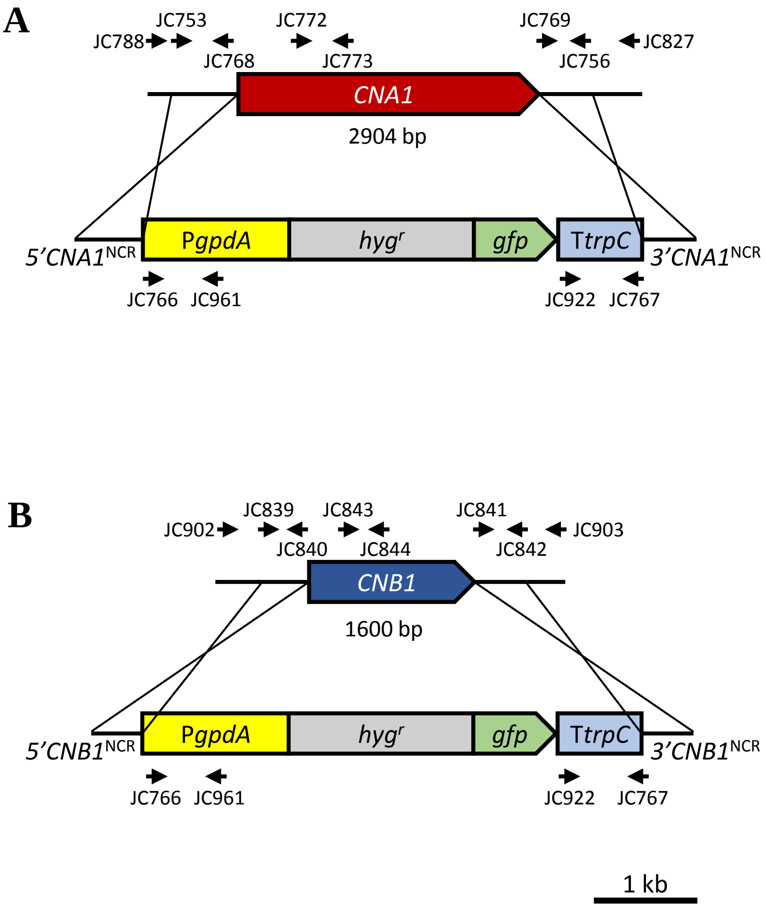


**Figure S3. Schematic diagram of *FolCNA1* and *FolCNB1* disruption construct.** A diagram showing targeted gene disruption in *Fol* non-coding region (NCR) flanking 5′ and 3′ ends of the *FolCNA1* **(A)** or *FolCNB1* **(B)** gene was fused with the hygromycin resistance cassette under the control of P*gpdA* promoter and T*trpC* terminator by fusion PCR, and further transformed into the protoplast of the wild type (4287) to carry out homologous recombination. Arrows represent primers used to amplify specific genes or to verify whether successful for targeted DNA replacement.

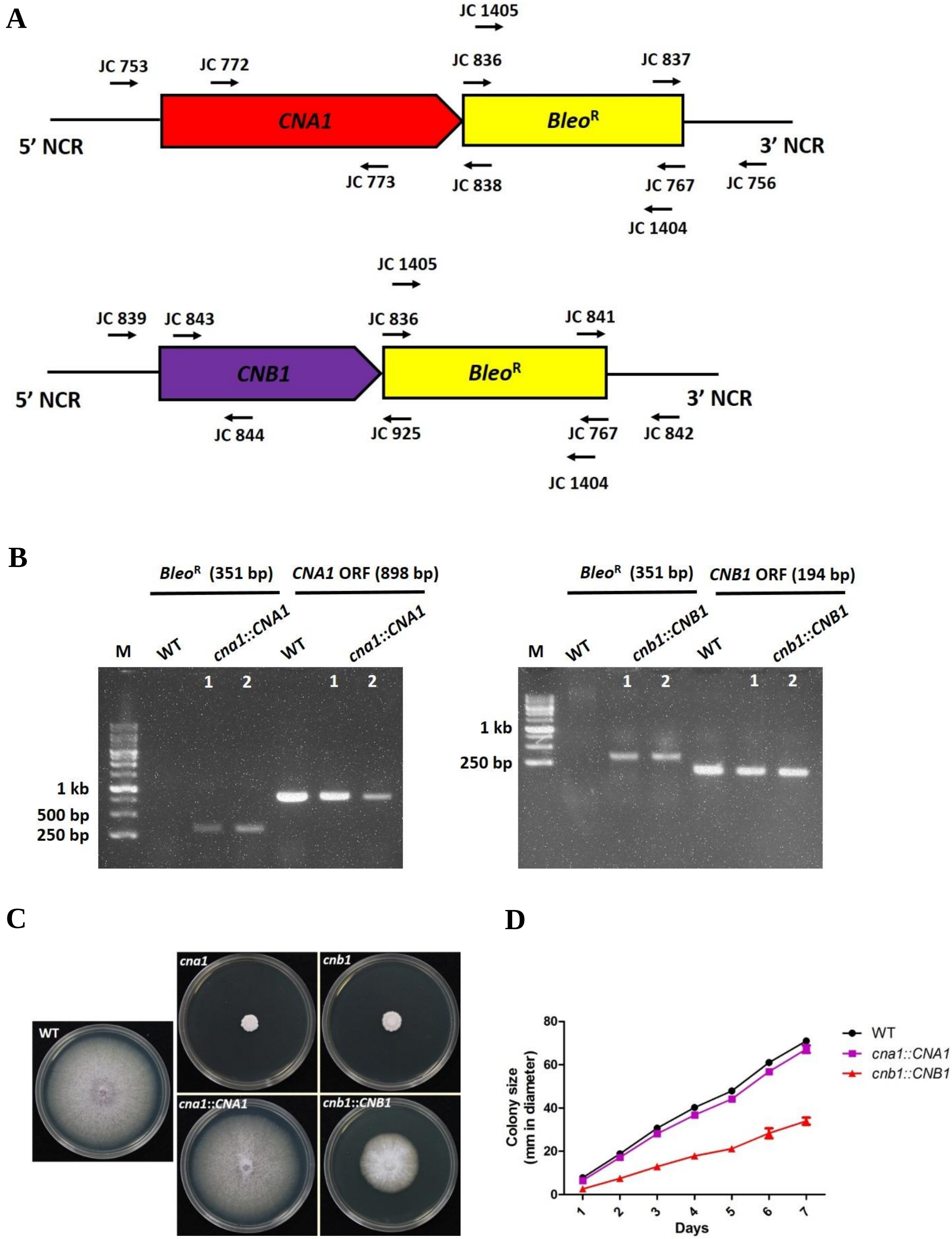

A
JC 1405
JC 753
JC 772
JC 836
JC 837
CNA1
BleoR
5’ NCR
3’ NCR
JC 773
JC 838
JC 767
JC 756
JC 1404
JC 1405
JC 839
JC 843
JC 836
JC 841
CNB1
BleoR
5’ NCR
3’ NCR
JC 844
JC 925
JC 767
JC 842
JC 1404
B
BleoR (351 bp)
CNA1 ORF (898 bp)
M
WT
cna1::CNA1
WT
cna1::CNA1
1
2
1
2
1 kb
500 bp
250 bp
BleoR (351 bp)
CNB1 ORF (194 bp)
M
WT
cnb1::CNB1
WT
cnb1::CNB1
1
2
1
2
1 kb
250 bp
C
WT
cna1
cnb1
cna1::CNA1
cnb1::CNB1
D
Colony size (mm in diameter)
80
60
40
20
0
1
2
3
4
5
6
7
Days
WT
cna1::CNA1
cnb1::CNB1

**Figure S4. The growth defects caused by the genetic deletion of *CNA1* and *CNB1* were restored in the complemented strains. (A)** Schematic diagram of *cna1* and *cnb1* complementation construct. A diagram showing *FolCNA1* or *FolCNB1* open reading frame (ORF) fused with the bleomycin resistance cassette by fusion PCR, and further transformed into the protoplast of *cna1* or *cnb1* mutant, respectively, to carry out homologous recombination. Arrows represent primers used to amplify specific genes or to verify whether successful for targeted DNA replacement. **(B)** PCR analysis was used to confirm the complementary strains (*cna1*::*CNA1* and *cnb1*::*CNB1*). Primers were listed in Table S1. **(C)** Radial growth of the wild type, calcineurin mutants, and complementary strains. All plates were incubated at 25°C for one week. **(D)** Growth kinetics of the wild type and complementary strains. Colony sizes of each strain were measured in diameter daily for 7 days. Error bars represent standard deviations of three technical replicates.

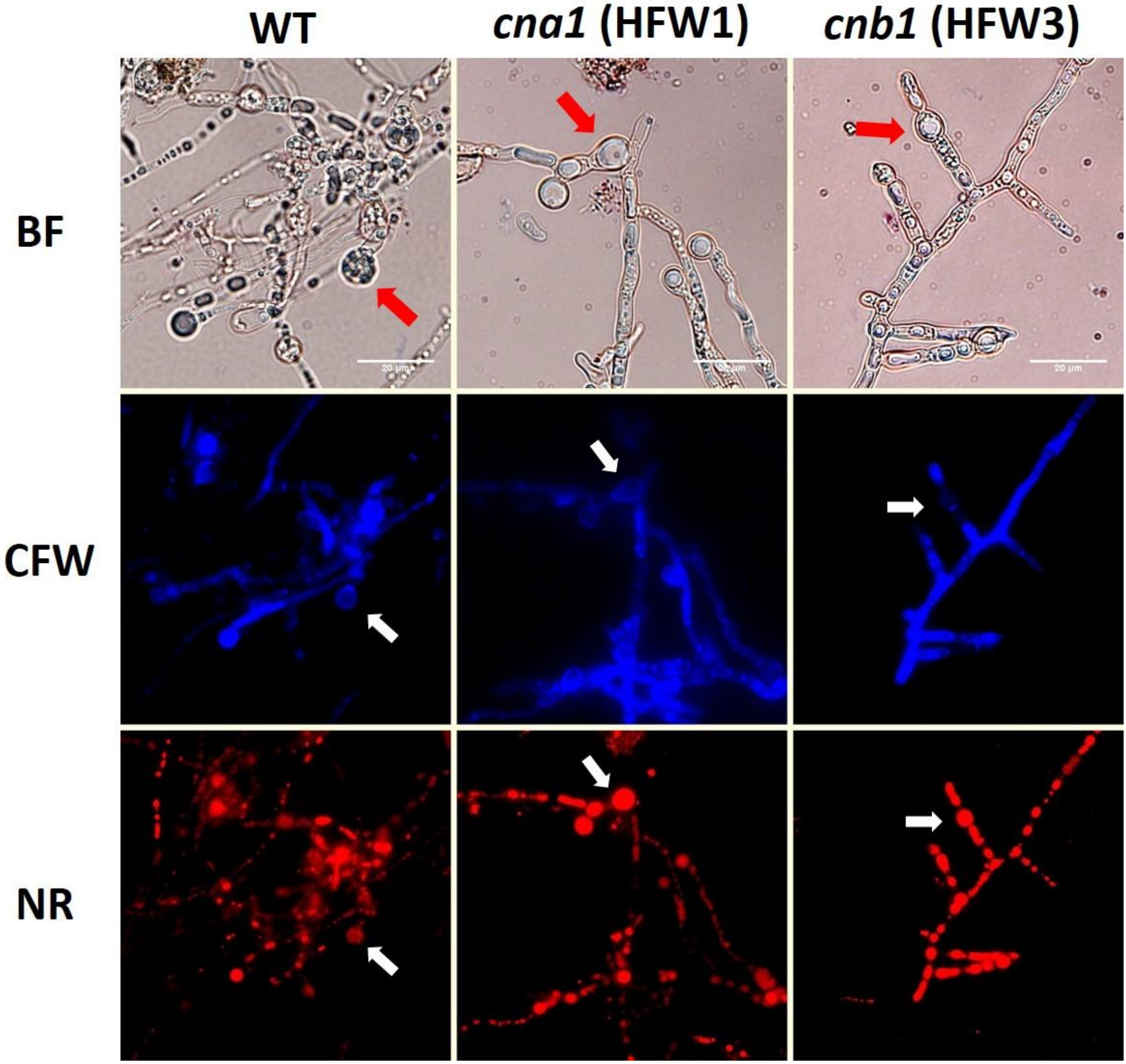


**Figure S5. Calcofluor white (CFW) and Nile red (NR) staining of *Fol* wild type and calcineurin mutants.** Conidia from wild type and calcineurin mutants were inoculated into soil extract to induce chlamydospore formation and then collected as described in Materials and Methods. Arrows indicate the chlamydospores. Scale bar, 20 µm.

**A**

dH$_2$O

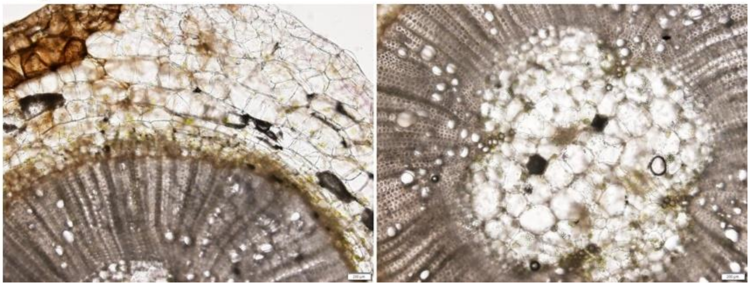

**B**

WT

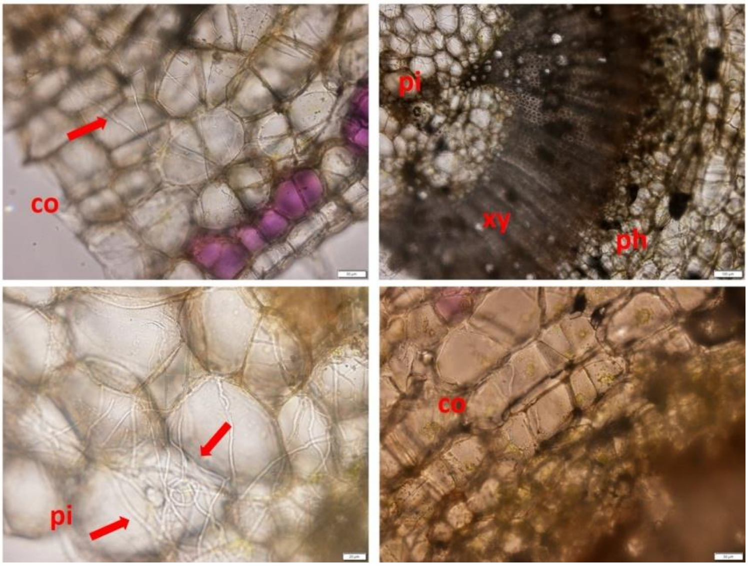


**C**

*cna1* (HFW1)

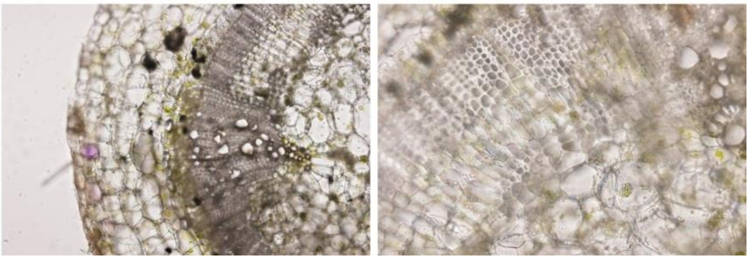

**D**

*cnb1* (HFW3)

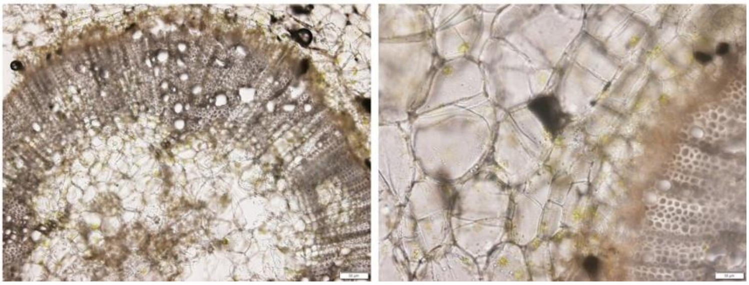

**Figure S6. Histological observation in the stem cross sections of tomato seedlings inoculated with the *Fol* wild type or calcineurin mutant.** The two-week-old tomato seedlings were inoculated with  a suspension of $5 \times 10^6$ conidia/mL of *Fol* wild type or calcineurin mutant by submerging tomato roots, and then transplanting into green house for 21 days. Cross section conducted at the stem 1 cm above the ground was examined and photographed. Tomato seedling inoculated with $dH_2O$. Scale bar, 200 μm **(A)**, *Fol* wild type. Scale bar, 200 μm **(B)**, *cna1* mutant. Scale bar, 200 μm (left) and 50 μm (right) **(C)**, or *cnb1* mutant. Scale bar, 50 μm **(D)**. Arrows indicate the infected hyphae constitute a cross network between cortex cells and cell surface, while inoculated with *Fol* wild type (B). Whereas no obvious hyphae can be found on the cortex cells or cell surface, while seedlings inoculated with $dH_2O$ or calcineurin mutant (A, C and D).  (pi: pith; co: cortex; xy: xylem; ph: phloem).

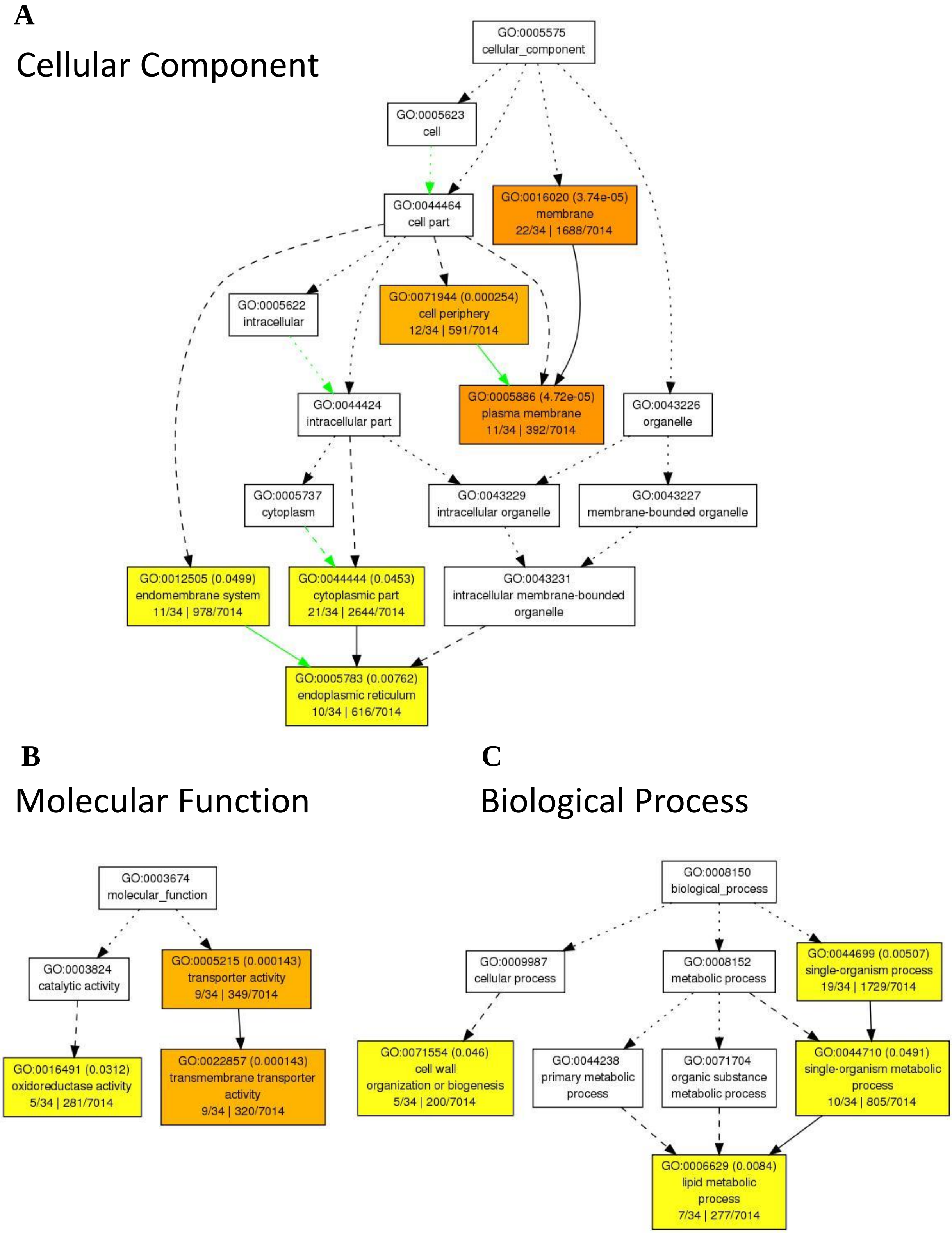

A
Cellular Component
GO:0005575
cellular_component
GO:0005623
cell
GO:0044464
cell part
GO:0016020 (3.74e-05)
membrane
22/34 | 1688/7014
GO:0005622
intracellular
GO:0071944 (0.000254)
cell periphery
12/34 | 591/7014
GO:0044424
intracellular part
GO:0005886 (4.72e-05)
plasma membrane
11/34 | 392/7014
GO:0043226
organelle
GO:0005737
cytoplasm
GO:0043229
intracellular organelle
GO:0043227
membrane-bounded organelle
GO:0012505 (0.0499)
endomembrane system
11/34 | 978/7014
GO:0044444 (0.0453)
cytoplasmic part
21/34 | 2644/7014
GO:0043231
intracellular membrane-bounded
organelle
GO:0005783 (0.00762)
endoplasmic reticulum
10/34 | 616/7014
B
Molecular Function
GO:0003674
molecular_function
GO:0003824
catalytic activity
GO:0005215 (0.000143)
transporter activity
9/34 | 349/7014
GO:0016491 (0.0312)
oxidoreductase activity
5/34 | 281/7014
GO:0022857 (0.000143)
transmembrane transporter
activity
9/34 | 320/7014
C
Biological Process
GO:0008150
biological_process
GO:0009987
cellular process
GO:0008152
metabolic process
GO:0044699 (0.00507)
single-organism process
19/34 | 1729/7014
GO:0071554 (0.046)
cell wall
organization or biogenesis
5/34 | 200/7014
GO:0044238
primary metabolic
process
GO:0071704
organic substance
metabolic process
GO:0044710 (0.0491)
single-organism metabolic
process
10/34 | 805/7014
GO:0006629 (0.0084)
lipid metabolic
process
7/34 | 277/7014

**Figure S7. Gene ontology enrichment analysis of the DEGs that coordinately regulated in both *cna1* and *cnb1* mutants.** Genes are functional correlated with the Cellular component **(A)**, Molecular function **(B)**, and **(C)** Biological process.

**A**

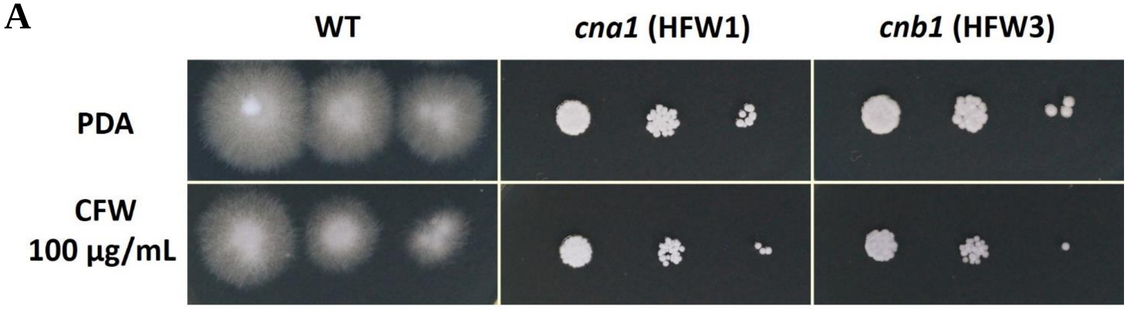


**B**

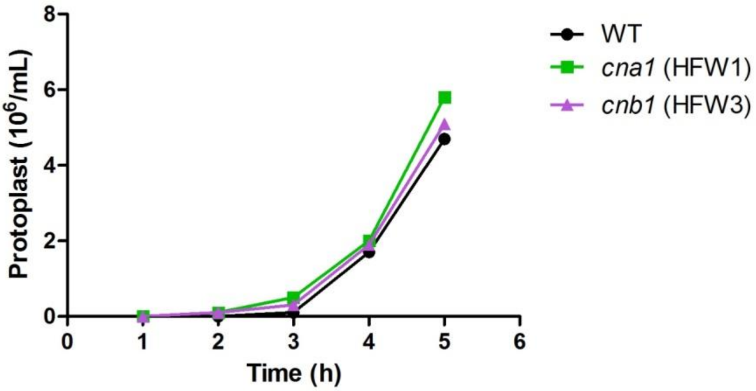


**Figure S8. The cell wall integrity analysis of *Fol* wild type and calcineurin mutants. (A)** The cell wall interference agent calcofluor white (CFW) can slightly reduce the colony growth of calcineurin mutants compared with the wild type. Vegetative growth of the wild type and calcineurin mutants was observed in the absence or presence of CFW. All plates were incubated at 25°C for 3 days and photographed. **(B)** The protoplast formation efficiency has no significant difference between the wild type and calcineurin mutants. The $10^8$ conidia/mL of the wild type and calcineurin mutants were inoculated in PDB media and incubated at 25°C for 16 h, then mycelia were collected and protoplasted with 10 mg/mL lysing enzyme. The protoplasts were counted using a hemocytometer.
